# FX06 to rescue SARS-CoV-2-induced acute respiratory distress syndrome: a randomized clinical trial

**DOI:** 10.1186/s13054-023-04616-1

**Published:** 2023-08-29

**Authors:** Emmanuelle Guérin, Lisa Belin, Guillaume Franchineau, Loïc Le Guennec, David Hajage, Mamadou Hassimiou Diallo, Thomas Frapard, Lucie Le Fèvre, Charles-Edouard Luyt, Alain Combes, Stéphane Germain, Jan Hayon, Pierre Asfar, Nicolas Bréchot

**Affiliations:** 1 Service de Médecine Intensive-Réanimation, Institut de Cardiologie, Assistance Publique–Hôpitaux de Paris (APHP), Hôpital Pitié–Salpêtrière, Paris, France; 2 Center for Interdisciplinary Research in Biology, Collège de France, Centre National de la Recherche Scientifique, Institut National de la Santé et de la Recherche Médicale (INSERM), Université PSL, Paris, France; 3 Sorbonne Université, INSERM, Institut Pierre Louis d’ Epidémiologie et de Santé Publique, AP-HP, Hôpital Pitié Salpêtrière, Département de Santé Publique, Unité de Recherche Clinique PSL-CFX, CIC-1901, 75013 Paris, France; 4 Intensive Care Unit, Centre Hospitalier Intercommunal de Poissy–Saint-Germain-en-Laye, Poissy, France; 5 INSERM U1018, Centre de Recherche en Épidémiologie Et Santé Des Populations (CESP), Equipe “Rein et Cœur”, Université Paris Saclay, Villejuif, France; 6 Médecine Intensive-Réanimation Neurologique, Hôpital Pitié–Salpêtrière, APHP, Paris, France; 7 Sorbonne Universités, Paris, France; 8 Sorbonne Université INSERM-UMRS 1166, Institute of Cardiometabolism and Nutrition, Paris, France; 9 Service de Médecine Intensive-Réanimation et Médecine Hyperbare, Centre Universitaire Hospitalier d’Angers, Angers, France; 10 Service de Médecine Intensive-Réanimation, Hôpital Européen Georges-Pompidou, APHP, Paris, France; 11 Université Paris Cité, Paris, France

**Keywords:** FX06, Vascular leakage, Endothelial hyperpermeability, Acute respiratory distress syndrome, SARS-CoV-2

## Abstract

**Background:**

Vascular leakage is a major feature of acute respiratory distress syndrome (ARDS). We aimed to evaluate the efficacy of FX06, a drug under development that stabilizes interendothelial cell junctions, at reducing vascular leakage during SARS-CoV-2-induced ARDS.

**Methods:**

This multicenter, double-blinded, randomized trial included adults with COVID-19-associated ARDS who had received invasive mechanical ventilation for < 5 days and were randomized to receive either intravenous FX06 (400 mg/d, for 5 days) or its vehicle as placebo. The primary endpoint was the lowering—from day 1 to day 7—of the transpulmonary thermodilution-derived extravascular lung-water index (EVLWi).

**Results:**

Twenty-five patients were randomized to receive FX06 and 24 the placebo. Although EVLWi was elevated at baseline (median [IQR] 15.6 mL/kg [13.5; 18.5]), its declines from day 1 to day 7 were comparable for FX06 recipients and controls (respectively, − 1.9 [− 3.3; − 0.5] vs. − 0.8 [− 5.5; − 1.1] mL/kg; estimated effect − 0.8 [− 3.1; + 2.4], *p* = 0.51). Cardiac indexes, pulmonary vascular permeability indexes, and fluid balances were also comparable, as were PaO_2_/FiO_2_ ratios and durations of mechanical ventilation. Adverse event rates were similar for the 2 groups, although more FX06 recipients developed ventilator-associated pneumonia (16/25 (64%) vs. 6/24 (24%), *p* = 0.009).

**Conclusions:**

In this unique-dosing–regimen study, FX06 did not lower SARS-CoV-2-induced pulmonary vascular leakage. Future investigations will need to evaluate its efficacy at earlier times during the disease or using other regimens.

*Trial registration* NCT04618042. Registered 5 November 2020.

**Supplementary Information:**

The online version contains supplementary material available at 10.1186/s13054-023-04616-1.

## Introduction

Vascular leakage is a major feature of pathogen-induced acute respiratory distress syndrome (ARDS) [1]. Triggered by inflammation following endothelial and epithelial lesions, it is thought to play an important role in altering gas exchanges. Consequently, the extravascular lung-water index (EVLWi), a marker of pulmonary vascular leakage measured by transpulmonary thermodilution, is independently associated with ARDS patients’ outcomes [2, 3]. Because EVLWi is highly elevated during SARS-CoV-2-induced ARDS [4, 5], which causes high mortality [4, 6], controlling vascular leakage might be of major interest in managing this disease.

FX06, an innovative drug containing fibrin-derived peptide Bβ15-42, stabilizes vascular endothelial (VE)-cadherin–dependent interendothelial cell junctions [7–9]. It reduced capillary leakage in several animal models of lipopolysaccharide- or HCl-induced acute lung injury [9, 10] and prolonged survival in a murine model of dengue-virus infection [9]. In a phase II trial conducted on 234 patients suffering from ischemia–reperfusion injuries during acute coronary syndrome, FX06-treated patients had 58% smaller early necrotic core zones [11]. Importantly, adverse events were comparable between groups, indicating the drug’s high safety profile. FX06 was then used as salvage therapy for a patient with severe ARDS following Ebola-virus infection, with a temporal link between its injection and sharply decreased EVLWi [12]. More recently, FX06 (400 mg/d for 4–7 days) was given as compassionate therapy to 6 patients receiving extracorporeal membrane oxygenation (ECMO) for coronavirus disease 2019 (COVID-19) [13]; 4 experienced improvement and 2 died. No clear treatment-related adverse event occurred.

Taken together, those findings indicate that FX06 is well-tolerated by patients and is a potent regulator of vascular leakage during ARDS. We hypothesized that FX06 might limit pulmonary vascular hyperpermeability during ARDS induced by SARS-CoV-2 infection, thereby improving gas exchanges and patients’ outcomes.

## Methods

### Trial design

We conducted a multicenter, double-blinded, randomized trial. The independent ethics review board CPP Ouest VI, Brest, France, and the ANSM (Agence Nationale de Sécurité du Médicament et des Produits de Santé) approved the trial protocol (available in  Additional file 1). F4-Pharma Ges.m.b.H. (Vienna, Austria) provided FX06. An independent Data- and Safety-Monitoring Committee periodically reviewed safety outcomes, with recruitment interruptions planned after inclusions of 10 and 30 patients. Neither F4-Pharma nor trial sponsors participated in the trial design, data collection, analysis or interpretation, or the writing or submission of the manuscript. The study protocol was registered at ClinicalTrials.gov (NCT04618042).

### Participants

To be eligible for inclusion, patients had to be ≥ 18 year old and receiving invasive mechanical ventilation for < 5 days for polymerase-chain reaction-confirmed SARS-CoV-2-induced ARDS, according to the Berlin definition [14]. Exclusion criteria were mechanical ventilation for > 4 days; participation in another interventional clinical trial; severe renal, hepatic or cardiac insufficiency, or in a moribund state at randomization (see Additional file 1); contraindication for vascular access implantation for transpulmonary thermodilution monitoring; chemotherapy, radiotherapy or immunotherapy for malignancy; pregnancy or lactation; any history of severe allergic drug reaction. Patients taking drugs interfering with inflammation were also excluded, unless the drug’s use during COVID-19 was stated in the hospital center’s written policy.

According to the specifications of emergency consent, randomization was possible without a close relative’s or surrogate’s consent, but informed consent by the patient or patient’s relatives was obtained for all patients.

### Treatment allocation

Patients were randomly assigned to receive either FX06 or its vehicle (phosphate-buffered saline) as the placebo. The randomization list was computer-generated with a 1:1 ratio and undisclosed block sizes, stratified by center. Concealment of the study-group assignments used a centralized, secure, interactive, web-based response system (CleanWeb, Telemedicine Technologies S.A.S., Boulogne-Billancourt, France) accessible from each study center. All investigators, statisticians, and data analysts were blinded to arm assignments until the study and analysis were completed.

### Interventions

Patients were randomized to receive intravenous FX06, 400 mg/d or the placebo for 5 days. Each dose was administered in two boluses separated by a 10-min interval. The dose regimen chosen was based on the results of previous studies, in animals and humans, that suggested safety and mechanistic engagement with this dosing (additional Methods in Additional file 2). The manufacturer provided each treatment in unrecognizable ready-to-use form (numbered and sealed therapeutic units containing 10 vials of active treatment or placebo solution), stocked in each intensive care unit (ICU) under the supervision of each facility’s pharmacy department.

Patients were monitored using transpulmonary thermodilution systems (EV1000/Volume View, Edwards Lifesciences, Irvine, CA, USA, or PiCCO2, Pulsion Medical Systems, Feldkirchen, Germany), with thermistor-tipped catheters introduced in a femoral artery and an internal jugular vein [2–4]. Extravascular lung water and other thermodilution-derived parameters were averaged from three injections of cold physiological saline solution, in supine position, and indexed to the patient’s predicted body weight. Thermodilution measurements were taken before treatment administration during the first 5 days post-inclusion, with a measurement repeated 3 h post-administration on day 2, to detect a possible short-time effect of the drug. For patients receiving venovenous (VV)-ECMO, measurements were taken during a transient diminution of ECMO blood flow to < 2 L/min. Preliminary study results showed that thermodilution parameters were not affected by ECMO blood flow under that level (see Additional file 2: Table S1 and Fig. S1).

For ARDS management, investigators were asked to follow the most recent recommendations from the French Society of Intensive Care Medicine (https://www.srlf.org/rfe-srlf-prise-en-charge-du-syndrome-de-detresse-respiratoire-aigue-sdra-de-ladulte-a-la-phase-initiale/). Specific treatments targeting COVID-19 were discouraged, unless the drug’s use during COVID-19 was stated in the center’s written policy.

Serum interleukin (IL)-6, IL-10, and soluble (s)VE-cadherin were quantified with DuoSet Elisa kits (R&D systems, Minneapolis, MN, USA).

### Outcomes

The primary endpoint was the EVLWi change, assessed by transpulmonary thermodilution, between day 1 and day 7. Secondary endpoints included the evolution of daily EVLWi, cardiac index, global end-diastolic volume index, and pulmonary vascular permeability index measured by transpulmonary thermodilution for 7 days; daily fluid balance; serum albumin; systolic, diastolic, and mean blood pressures; and heart rate for 7 days; partial oxygen pressure/fraction of inspired oxygen (PaO_2_/FiO_2_) ratio and Sequential Organ-Failure Assessment (SOFA) score over 15 days; rate of rescue with VV-ECMO; durations of invasive mechanical ventilation, vasopressor support, and renal replacement therapy over 30 days; Weinberg Radiological Severity score over 30 days [15]; survival at 30 and 60 days; nature and frequency of adverse events. Kinetics of serum d-dimers and C-reactive protein over 7 days were extracted from medical charts afterward. Serum IL-6, IL-10, and sVE-cadherin measurements on days 1 and 7 in available biological samples were added as post hoc measurements.

### Statistical analyses

Assuming a baseline (inclusion) mean EVLWi of 13 mL/kg and standard deviation (SD) of 5 mL/kg [16], and a 30% EVLWi decrease in FX06-treated patients compared to controls on day 7 [9, 10, 12], for 80% power and an overall 5% two-sided α-risk, the required sample size was 25 patients/group.

Baseline characteristics are reported as number (%) for categorical variables and median [interquartile range, IQR] for continuous variables. Efficacy endpoints were analyzed according to intention-to-treat principles. Safety endpoints were analyzed for all patients who received at least one assigned-treatment dose.

Missing primary endpoints were replaced by imputation values for patients who died or whose conditions no longer warranted the transpulmonary thermodilution system before 7 days; the last thermodilution value was retained for the primary analyses. Primary endpoints were compared between groups using an adjusted analysis of covariance (ANCOVA) of EVLWi at randomization. Results are expressed in terms of adjusted mean change with 95% confidence interval (CI).

Three sensitivity analyses were computed: complete case analysis, worst-case analysis or using a different statistical method (Mann–Whitney U test). Prespecified subgroup analyses were conducted according to VV-ECMO or EVLWi > 10 mL/kg at inclusion.

Qualitative and quantitative secondary outcome measures were compared between groups using, respectively, Pearson’s Chi-square tests and *t* tests, or Mann–Whitney U tests. Overall survival was estimated with the Kaplan–Meier method. Longitudinal quantitative endpoints were compared using linear-mixed models with a random effect for subjects. This model was fitted to fixed effect by an interaction between treatment arm and time (since the date of randomization), with the slope parameter estimating the difference between groups. A restricted likelihood maximization-estimation method was used. The *p*-values associated with the fixed effects were calculated using the analysis of variance (ANOVA) function with Kenward–Roger approximation for calculating the number of degrees of freedom.

Analyses were computed with a 2-sided α risk of 5%. All analyses were performed using R software (R Foundation for Statistical Computing, Vienna, Austria), version 4.0.3.

## Results

**Fig. 1:**
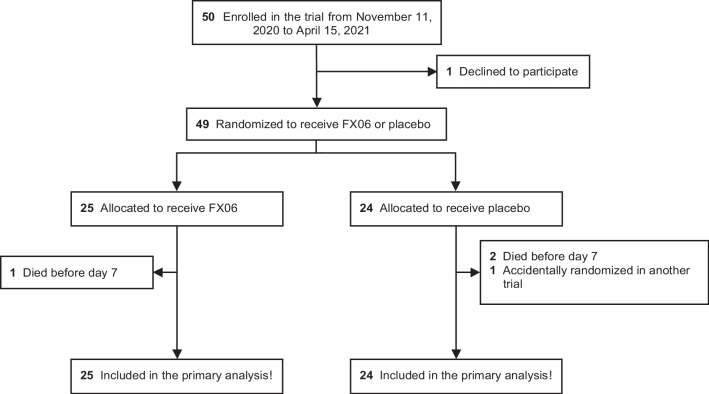
Flow chart of inclusion, randomization, and follow-up of patients included in this clinical trial

Forty-nine patients were randomized from November 2020 to April 2021 and retained for analysis (Fig. 1). Their main characteristics are reported in Table 1. They were very severely ill at baseline, with median PaO_2_/FiO_2_ ratio at 104, static pulmonary compliance of < 30 mL/cmH_2_O, and more than one-third of them were on VV-ECMO. One-third were receiving vasopressors. Specific therapies targeting COVID-19 were marginal, except corticosteroids, given to all participants.

**Table 1:** Characteristics of patients with COVID-19-associated ARDs at trial inclusion, according to assigned treatment arm

Characteristic	FX06 (*n* = 25)	Placebo (*n* = 24)
Age, y	59 [53; 67]	59 [53; 68]
Sex		
Male	19 (76)	16 (67)
Female	6 (24)	8 (33)
Pre-existing condition		
Diabetes	9 (36)	6 (25)
Hypertension	11 (44)	14 (58)
Coronary artery disease	4 (16)	2 (8)
Severe cardiovascular disease*	0	0
Chronic respiratory disease	1 (4)	2 (8)
Kidney disease†	1 (4)	0
Chronic liver disease	0	0
Immunodeficiency	0	0
Cancer	0	3 (12)
Body mass index, kg/m^2^	32 [28; 35]	30 [27; 36]
Simplified Acute Physiology Score II	58 [41; 65]	56 [38; 66]
Days to inclusion		
From symptom onset	14 [10; 18]	12 [9; 14]
From first positive PCR	12 [10–16]	9 [7–14]
From oxygen therapy onset	5 [1–7]	3 [2–5]
From invasive mechanical ventilation onset	2 [2; 4]	2 [2; 3]
COVID-19 therapy use		
Corticosteroids	25 (100)	24 (100)
Remdesivir	0	0
Immunomodulator (tocilizumab or sarilumab)	3 (12)	3 (12)
Other	0	0
Respiratory support prior to intubation		
High flow oxygen device	24 (96)	19 (79)
High flow oxygen device duration, days	2.5 [0.25–5]	2 [1–2.75]
Non-invasive mechanical ventilation (NIV)	8 (32)	6 (25)
NIV duration, days	2 [1–3]	1.5 [0–4.5]
Respiratory status		
Tidal volume, mL/kg of PBW	3.7 [2.7; 4.7]	4.4 [3.0; 4.8]
Plateau pressure, cmH_2_O	26 [24; 28]	25 [24; 27]
Positive end-expiratory pressure, cmH_2_O	12 [12; 15]	12 [10; 14]
Cst_rs_‡, mL/cmH_2_O	26.2 [18.7; 29.4]	27.8 [21.1; 38.7]
FiO_2_	100 [60; 100]	100 [64; 100]
VV-ECMO	10 (40)	7 (29)
Arterial blood gases		
PaO_2_, mmHg	68 [60; 92]	82 [70; 96]
PaO_2_/FiO_2_§, mmHg	102 [62; 145]	110 [73; 187]
PaCO_2_, mmHg	42 [39; 47]	42 [39; 44]
Arterial pH	7.43 [7.40; 7.46]	7.40 [7.39; 7.46]
Vasopressor use	9 (36)	9 (37)
Serum creatinine, μmol/L	66 [51; 86]	66 [46; 97]
C-reactive protein, mg/L	102 [58; 159]	120 [79; 215]
Fibrinogenemia, g/L	6.6 [5.8; 7.4]	7.0 [6.0; 8.4]
d-Dimers, mg/L	2650 [1029; 8348]	2625 [1335; 4872]

Values are expressed as *n* (%) or median [IQR 25th; 75th percentiles]. *NIV* non-invasive mechanical ventilation; *PBW* predicted body weight; *VV-ECMO* venovenous extracorporeal membrane oxygenation

^*^Defined as New York Heart Association class III or IV. †Determined from the most recent stable serum creatinine level prior to this hospital admission, except for patients on dialysis. Abnormal kidney function was defined as serum creatinine ≥ 130 μmol/L (≥ 1.5 mg/dL) for males or ≥ 100 μmol/L (≥ 1.1 mg/dL) for females not previously on dialysis. ‡Cst_rs_ static compliance of the respiratory system; Cst is the tidal volume in mL divided by the driving pressure (plateau pressure—positive end-expiratory pressure) in cmH_2_O §For VV-ECMO patients, last ratio before ECMO implantation

### Study drug

All but 5 patients received the complete treatment. One patient allocated to receive the placebo was accidentally included in another interventional study; the assigned treatment was interrupted after 3 days. One patient allocated to the FX06 arm stopped treatment after 3 days because of fungal co-infection. Two patients allocated to the placebo arm died on day 3 or day 4. Lastly, one patient’s treatment was accidentally withheld on day 4.

### Primary outcome

**Fig. 2:**
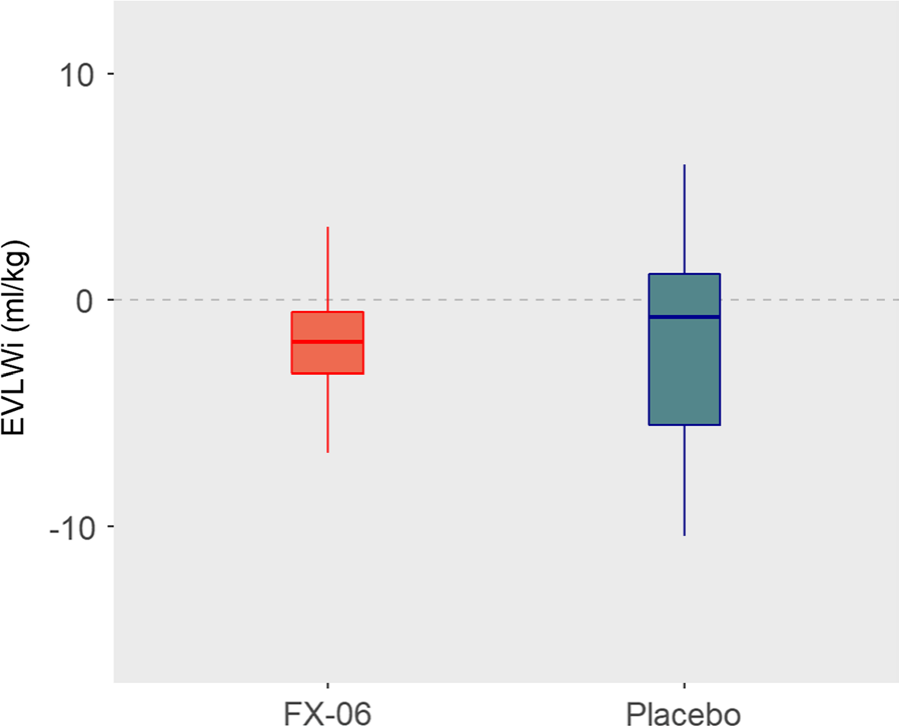
Primary outcome: extravascular lung-water index (EVLWi) variations from day 1 to day 7 for FX06 recipients and placebo controls. The panels show Tukey’s boxplot visualization

The primary outcome of EVLWi change between days 1 and 7 did not differ between FX06-treated patients and controls (Fig. 2 and Table 2). EVLWi kinetics and their individual variations were also comparable between groups (Additional file 2: Figs. S2 and S3). Patients’s EVLWis were high at inclusion, comparable for the 2 groups, and remained elevated during the first 7 days. Eight patients—1 FX06 recipient and 7 controls—did not undergo transpulmonary thermodilution on day 7; their last available values were retained for the primary analysis: 3 had died (1 FX06 recipient and 2 controls), 3 had recovered sufficiently to allow removal of their thermodilution catheters, 1 had a catheter infection necessitating its removal, and 1 withdrew consent to participate. Excluding those patients did not affect the primary-analysis results.

**Table 2:** Primary and secondary outcomes according to assigned treatment arm

Characteristic	FX06	Placebo	Estimated effect* (95% CI)	*p*-value
D1–D7 EVLWi variation†, mL/kg	− 1.9 [− 3.3; − 0.5]	− 0.8 [− 5.5; 1.1]	− 0.48 [− 3.2; 2.3]	0.72
EVLWi†, mL/kg				
D1	15.9 [13.7; 18.3]	15.5 [12.6; 18.6]		0.38
D7	14.4 [12.3; 17.5]	12.4 [10.0; 17.2]		0.89
Survival				
D7	24/25 (96)	21/24 (88)	− 8% [− 10%; 29%]^ʊ^	0.35
D30	21/25 (84)	17/24 (71)	− 13% [− 37%; 11%]^ʊ^	0.27
D60	17/25 (68)	17/24 (71)	+ 3% [− 24%; 30%]^ʊ^	0.83
D30 respiratory support-free days‡	0 [0; 14]	9 [0; 19]	OR = 0.4 [0.1; 1.2] /IRR = 1.1 [0.8; 1.5]	0.19
D30 vasopressor-free days§	18 [12; 29]	17 [0; 30]	1 [− 15; 20]	0.90
D30 RRT-free days¶	30 [23; 30]	30 [0; 30]	0 [− 12; 9]	0.49
D30 SOFA-free days||	0 [0; 0]	0 [0; 1]	OR = 0.4 [0.1; 1.5] /IRR = 1.1 [0.3; 4.4]	0.41
Plasma d-dimer, ng/L			− 358 [− 1120; 634]^ϕ^	1.0
D1	2650 [1029; 8348]	2625 [1335; 4872]		
D7	3190 [1691; 8360]	2270 [1339.5; 3544]		
Serum C-reactive protein, mg/L			− 48 [− 118; 53]^ϕ^	0.90
D1	102 [57.8; 159]	120 [79; 215]		
D7	124 [87; 185]	140 [79; 193]		
Plasma IL-6**, pg/mL			29 [− 125; 115]^ϕ^	0.91
D1	24.9 [15.5; 93.6]	23.4 [3.85; 42.1]		
D7	118.2 [41.8; 218.7]	25.2 [14.9; 153.3]		
Plasma IL-10**, pg/mL			0 [− 188; 6]^ϕ^	0.93
D1	0.2 [0; 34.9]	2.1 [0; 52.6]		
D7	8.1 [0; 25.7]	0.5 [0; 69.0]		
Plasma sVE-cadherin**, ng/mL			38 [− 170; 195]^ϕ^	0.57
D1	586.3 [540.1; 681.6]	473.5 [393.1; 570.9]		
D7	628.7 [569.4; 793.1]	676.6 [580.5; 692.1]		

Values are expressed as *n* (%) or median [25th; 75th percentiles, IQR]. D day. *Between-group difference of the mean variation of extravascular lung-water index (EVLWi) from day 1 to day 7, adjusted to the initial EVLWi. †Extravascular lung-water indexed to the predicted body weight. ‡Number of days alive without invasive mechanical ventilation. The effect estimate from Hurdle model is odds ratio to have more than 0 days alive without invasive mechanical ventilation/incidence risk ratio and bootstrap 95% CI. §Number of days alive without vasopressors. The effect estimate was measured with a median difference between arms and bootstrap 95% CI. ¶Number of days alive without renal replacement therapy. The effect estimate was measured with a median difference between arms and bootstrap 95% CI. ||Number of days alive without organ failure, defined by one or more Sequential Organ-Failure sub-Scores ≥ 3. The effect estimate obtained from Hurdle model is incidence risk ratio to have more than 0 days alive without organ failure/incidence risk ratio and bootstrap 95% CI. **Available for 13 FX06 recipients and 11 controls. ^ʊ^the effect estimate was measured with a difference of proportion between arms and its exact binomial 95% confidence interval. ^ϕ^The effect estimate was measured as a median difference of change from baseline between arms and bootstrap 95% CI

Several sensitivity analyses were computed: analyzing the primary outcome for patients with ECMO vs without; most severely ill (EVLWi > 16 mL/kg) vs less severely ill; or indexing the dose received above vs below 4.2 mg/kg/d (median dose received). All failed to detect any significant FX06 effect on EVLWi (not shown). Interestingly, EVLWi measured before and 3 h after FX06 bolus injections was very similar (median variation − 0.02 [IQR − 0.57; 0.55], *n* = 22).

### Secondary outcomes

The Pulmonary Vascular Permeability Index (PVPI) was also very high during the first 7 days and did not differ between groups (Additional file 2: Fig. S4). Cardiac index and global end-diastolic volume index (GEDVI) were also comparable (not shown). Daily fluid balance remained positive during the first week, with comparable levels for the 2 groups (Additional file 2: Fig. S5). Serum albumin, another marker of vascular leakage, was very low at inclusion (median 23 [IQR 19; 26] g/L; *n* = 48); it remained stable with no between-group differences during the first week.

PaO_2_/FiO_2_ remained very low during the first 15 days (Additional file 2: Fig. S6), and very few patients survived to be extubated on 30 days (Table 2). Interestingly, the Weinberg Radiological Severity score decreased less for FX06 recipients (estimated effect 0.13 [95% CI 0.07–0.18]; *p* < 0.001). However, for a post hoc analysis taking into account missing values and mortality as a competing risk by imputing the last score available for survivors and a score of 12 after the patient died, score kinetics did not differ between groups (Additional file 2: Fig. S7). Three patients (2 FX06 recipients and 1 control) received VV-ECMO rescue therapy after inclusion.

Although catecholamine-free days were comparable for the 2 groups (Table 2), FX06 recipients had significantly lower systolic and diastolic blood pressure values after day 4 (Additional file 2: Fig. S8). FX06 did not affect heart rate (not shown).

The baseline SOFA score, reflecting the extent of multiple organ failure, was high and remained stable for both groups for 15 days (Additional file 2: Fig. S9). Finally, survival was comparable for both groups, with 34/49 (69%) day-60 survivors (Table 2 and Additional file 2: Fig. S10). C-reactive protein and serum d-dimers, elevated at baseline, remained stable and comparable. Serum cytokine measurements were available for 24 patients. For both groups at inclusion, IL-6 was elevated and continued to rise during the first week, while IL-10 levels were also high and declined slightly during the first week. FX06 recipients had slightly higher baseline sVE-cadherin values that remained comparable to those of the placebo group thereafter.

### Safety

Adverse event rates were comparable for the 2 groups (Table 3). Although overall secondary infections were not more common in FX06 recipients, they did develop more episodes of microbiologically confirmed ventilator-associated pneumonia [17].

**Table 3:** Serious adverse events (SAEs) reported according to assigned treatment arm

Serious adverse events	FX06	Placebo	*p*-value
Patients with at least one, *n* (%)	22/25 (88)	21/24 (88)	1.0
Number	94	72	0.36
SAE type*			
VV-ECMO rescue	2	1	0.59
Infectious event	22	18	0.11
Bacteriemia	4	9	0.11
Ventilator-associated pneumonia, *n* (%)	16 (64%)	6 (24%)	0.009
Septic shock	2	2	1.0
Ischemic stroke	1	0	1.0
Hemorrhagic stroke	0	2	0.23
Cardiac rhythm disorder	0	2	0.23
Cardiogenic shock	0	1	0.49
Gastrointestinal ulcer	1	0	1.0
Liver cytolysis/cholestasis	1	3	0.35
Acute kidney injury	4	4	1.0
Anemia	1	0	1.0
Thrombocytopenia	0	2	1.0
Bleeding episode	3	2	1.0
Thrombosis	0	2	1.0
Cutaneous reaction	1	0	1.0
Rhabdomyolysis	0	1	0.49

Values are expressed as number unless stated otherwise. *VV-ECMO* Venovenous extracorporeal membrane oxygenation. *According to the Common Terminology Criteria for Adverse Events (CTCAE) v.4.0

## Discussion

In this multicenter, double-blinded, randomized trial, FX06 did not alter the thermodilution-measured EVLWi evolution during SARS-CoV-2-induced ARDS. Other markers of pulmonary vascular leakage, e.g., patients’ functional outcomes reflecting pulmonary function and 60-day survival, were also not affected. Although elevated at baseline, circulating markers of inflammation and endothelial lesions were comparable for the 2 groups. Despite their similar rates of serious adverse events, FX06 was associated with higher rates of ventilator-associated pneumonia.

Inflammation-induced pulmonary vascular leakage is widely diffused during severe SARS-CoV-2 infection. Autopsies of COVID-19 patients revealed markedly elevated lung weights [18, 19] and disruption of interendothelial VE-cadherin-dependent junctions [20]. Furthermore, COVID-19 patients’ plasmas were able to trigger rapid and sustained enhanced permeability of human pulmonary microvascular endothelial cells (HPMVEC) cultured in vitro [21]. Our patients’ baseline EVLWi values were very high, confirming extensive vascular hyperpermeability. C-reactive protein and IL-6 levels further confirmed high levels of inflammation. VE-cadherin-dependent vascular leakage, the mechanism targeted by FX06, was thus likely involved in our patients’ pulmonary lesions.

However, several factors limit the interpretation of our results. SARS-CoV-2 is responsible for a particular form of ARDS, in which several mechanisms other than VE-cadherin-mediated endothelial barrier disruption could account for vascular leakage that might have masked the FX06 effect. Results of autopsy series previously showed disruption of endothelial tight junctions [20]. They also revealed severe endothelial cell injury, with images of cell apoptosis and membrane disruption [18, 22]. In vitro study observations confirmed the cytotoxicity of plasmas from SARS-CoV-2-infected patients on endothelial cell monolayers [23]. Importantly, all autopsy findings highlighted widespread microvascular thromboses in the lungs [18, 20, 22] that might be responsible for disseminated endothelial cell dysfunction, lysis, and death [24]. Thus, other ARDS forms not originating from COVID-19 might be more suitable for FX06 evaluation, as they may be associated with less pronounced non-inflammatory physiological processes implicated in vascular leakage.

Our population had other limitations hampering treatment evaluation. With a median of 13 days between first symptoms and treatment initiation, it is likely that we missed the initial exudative phase of ARDS. That supposition is supported by the elevated EVLWi in our population at baseline. Notably, pulmonary edema remains a dynamic balance between fluid extravasation and resorption. Likewise, VE-cadherin-dependent interendothelial cell-junction stability relies on a dynamic balance between its internalization and recycling to the membrane [25]. Thus, by stabilizing VE-cadherin homotypic interactions, FX06 might still have had a net effect on extravascular fluid balance, even at late stages of ARDS. Such a late effect was suggested by the first FX06 rescue therapy during ARDS in humans, which occurred after 11 days of Ebola-virus infection [12]. However, we cannot exclude a more powerful FX06 effect in preventing vascular leakage at earlier stages.

Better characterization of vascular leakage kinetics during ARDS in humans and drug evaluation earlier during disease evolution are needed to determine the optimal window for its administration during ARDS. FX06 assessment in other entities with inflammation-induced vascular leakage, that would allow its prompt administration earlier during the disease course, might also be highly contributive. In this setting, post-resuscitation syndrome, in which the drug was highly effective in pre-clinical studies [26], might be of particular interest. Moreover, with one-third of the patients on VV-ECMO, a median PaO_2_/FiO_2_ ratio ~ 100 mmHg, and median static compliance of 27 mL/cmH_2_O, our population might have been too severely ill to detect an effect. Evaluating FX06 in less severe forms of COVID-19 remains of interest. Lastly, all of our patients were receiving corticosteroids, as recommended after the RECOVERY trial [27], which might have partially dampened inflammation, thereby masking the FX06 therapeutic effect.

The dosing regimen used is also open to debate. At a median of 4.2 mg/kg/d, our FX06 dose was close to the optimal one described for animals [9, 28], and those used as rescue therapy in humans [12, 13]. Although indexing the dose received to patients’ weight did not change the primary endpoint herein, we cannot exclude that higher doses might have achieved a detectable effect. Likewise, the FX06 half-life in the plasma of healthy volunteers was short (11–17 min) [29], even though FX06 effects linked to VE-cadherin persisted for hours or days in animal models [9, 10]. No EVLWi change was detected shortly (3 h) after its bolus injections in our study, which does not exclude that dosing regimens with repeated or continuous injections or higher doses might be more effective. In this context, PK/PD analyses of FX06 administration to critically ill patients should be highly informative. Although initially planned in our study, that analysis could not be done for technical reasons, in the context of the pandemic. Taken together, our data highlight the need for careful reassessment of the FX06-dosing regimen in future studies on humans.

Beyond PK, evaluating FX06 biological activity is complex. Although thermodilution-measured EVLWi accurately reflects the level of pulmonary edema [30], the hypothesized effect of 30% reduction in 7 days in FX06-treated patients might have been too large. FX06 limited vascular leakage by 60–80% in animal models of ARDS [9, 10] and was associated with 52% EVLWi reduction in a patient treated for Ebola-induced ARDS [12]. However, the multiplicity of factors impacting this parameter in humans might suggest a more restrictive approach to its anticipated reduction in future studies.

Other techniques might also be more sensitive in detecting vascular leakage and serve as better surrogate markers for FX06 development. Among markers implicated in endothelial physiology, several have been associated with patients’ outcomes after various causes of critical illnesses [31–33], including COVID-19 [34–36]. Among them, angiopoietin-2 might have the strongest association with the level of vascular leakage [32, 33]. We used sVE-cadherin as a possible surrogate marker of VE-cadherin stabilization by FX06. Although samples were only available for 24 patients, its serum concentration on day 7 was unaffected by FX06. However, despite sVE-cadherin being a marker of endothelial barrier disruption [37], its accuracy in predicting vascular leakage and patients’ outcomes remains debated [34, 38]. Whether other markers of endothelial injury might have been more sensitive in detecting an FX06 effect in our study is unknown and is currently the focus of ongoing studies. Whether some might also help improve patient selection for future studies remains to be seen.

VV-ECMO, implanted in one-third of our patients, may have also interfered with the evaluation of an FX06 effect, with the device influencing drug PK. However, considering the low lipophilic properties of FX06, significant adsorption onto the membrane is unlikely. EVLWi evaluation itself is also affected by VV-ECMO blood flow [39, 40]. Our observations in a preliminary study conducted on 20 patients at the time of ECMO explantation (Additional file 2: Methods), combined with the results of another group in 7 patients [39], showed that EVLWi determination is minimally affected for blood flows of < 2 L/min, like those used herein. Excluding patients on ECMO from the analysis also did not modify the results. Theoretically, pulmonary vascular obstruction might also contribute to EVLWi underestimation. Our COVID-19 patients, particularly at risk for pulmonary embolism, were not routinely screened for it. Although pulmonary embolism events did not affect the results of thermodilution-derived EVWLis in a recent study on COVID-19 patients [4], we cannot exclude they might have interfered with our evaluations.

Our observations on FX06 effect on arterial pressure are noteworthy. After 4 days of treatment, FX06 recipients were indeed less hypertensive than patients given the placebo. Although that finding remains exploratory, it might indicate an FX06 effect on the vasculature to be investigated in future studies.

With comparable rates of serious adverse events reported for both trial groups, our results confirm the good safety profile of FX06. Although exploratory, the higher rates of ventilator-associated pneumonia episodes developing in FX06 recipients warrant attention. Fibrin fragment Bβ15-42 was shown to inhibit leukocyte transendothelial migration in vitro [7] and to dampen neutrophil recruitment in the lung in two models of lipopolysaccharide- or HCl-induced acute lung injury, indicating a risk of impaired bacterial clearance [10]. Importantly, incubation of FX06 with monocytes or alveolar macrophages did not impact their in vitro activation and capacity to release pro-inflammatory cytokines. Moreover, on the contrary, FX06 was shown to enhance bacterial clearance and ultimately survival in a model of secondary *Pseudomonas aeruginosa* infection [10]. Although this potential effect of FX06 needs to be better clarified, these findings indicate the need for close monitoring of secondary infections in future evaluations of it.

## Conclusions

FX06 did not lower thermodilution-derived EVLWi during severe ARDS induced by SARS-CoV-2 infection. Whether other time-lines for its administration or other dosing regimens might be more efficient remains to be determined.

### Supplementary Information

**Additional file 1**. The study protocol.

**Additional file 2**. The supplementary data.

## Data Availability

All deidentified individual participant’s data collected for our study “FX06 to rescue acute respiratory distress syndrome during COVID-19 pneumonia. A randomized clinical trial” will be shared beginning with publication with no end date. These data will be available to researchers to who provide a methodologically sound proposal for the purposes of achieving specific aims outlined in that proposal. Proposals should be directed to the corresponding author via email: nicolas.brechot@aphp.fr and will be reviewed by the senior authors of the study. Requests to access data to undertake hypothesis-driven research will not be unreasonably withheld. To gain access, data requesters will need to sign a data-access agreement and to confirm that data will be used only for the agreed purpose for which access was granted.

## Abbreviations

ARDS: Acute respiratory distress syndrome
ECMO: Extracorporeal membrane oxygenation
EVLWi: Extravascular lung-water index
(s)VE-cadherin: (soluble) vascular endothelial cadherin
SARS-CoV-2: Severe acute respiratory syndrome–coronavirus-2
COVID-19: Coronavirus disease 2019
