# Supplementary material for: FX06 to rescue SARS-CoV-2-induced acute respiratory distress syndrome: a randomized clinical trial
Source: Crit Care. 2023 Aug 29;27:331. doi: 10.1186/s13054-023-04616-1 (PMC10463389; doi:10.1186/s13054-023-04616-1)
Supplement: Supplementary file 1 — Additional file 1. The study protocol. [file 13054_2023_4616_MOESM1_ESM.pdf]

FX06 to rescue acute respiratory distress syndrome during Covid-19 pneumonia
FX-COVID

INTERVENTIONAL RESEARCH PROTOCOL INVOLVING HUMAN PARTICIPANTS CONCERNING A MEDICINAL PRODUCT FOR HUMAN USE

Version N°3.0 of 22/03/2021

Project Code: APHP200495 / EUDRACT no: 2020-002056-20

Coordinating Investigator:
Nicolas BRECHOT
Medical-Surgical ICU
La Pitié-Salpêtrière hospital, Paris
Tel. +33-1-42-16-38-35
Email: nicolas.brechot@aphp.fr

Scientific Director:
David HAJAGE
Département Biostatistique, Santé Publique et Information Médicale - Unité de Recherche Clinique
Hospital Pitié Salpêtrière - AP-HP. Sorbonne Université
Tel +33-1-42-16-05-53 Email : david.hajage@aphp.fr

Sponsor :
Assistance Publique – Hôpitaux de Paris (AP-HP) and by delegation : Direction de la Recherche Clinique et de l'Innovation - DRCI (Clinical Research and Innovation Department)
Hôpital Saint-Louis - 1, avenue Claude Vellefaux
DRCI-Head Office project advisor: Carla VANDENABELE
Email: carla.vandenabele@aphp.fr

Entity responsible for monitoring the study:
Unité de Recherche Clinique (URC)-Pitié-Salpêtrière – Head : Pr. Florence TUBACH
Hospital Pitié-Salpêtrière - AP-HP. Sorbonne Université
47-83 Boulevard de l'Hôpital – 75651 Paris Cedex 13
Project advisor: Jessica PALMYRE Email: Jessica.palmyre@aphp.fr

Safety contact:
Secteur Vigilance – DRCI - Hôpital Fernand Widal 6 - 200, rue du Faubourg Saint-Denis - 75475 PARIS
Dr Sarra DALIBEY Email: sarra.dalibey@aphp.fr
Dr Hélène BROCVIELLE Email: helene.brocvielle@aphp.fr

Entity responsible for the pharmaceutical organization and coordination
Agence générale des équipements et produits de santé (AGEPS) (APHP)
Département Essais Cliniques (DEC) - 7, rue du Fer à Moulin, 75005 Paris
Dr Annick TIBI E-mail: annick.tibi@aphp.fr

SIGNATURE page for a research PROTOCOL

INTERVENTIONAL RESEARCH PROTOCOL INVOLVING HUMAN PARTICIPANTS CONCERNING A MEDICINAL PRODUCT FOR HUMAN USE

Research code number: APHP200495

Title: FX06 to rescue acute respiratory distress syndrome during Covid-19 pneumonia - **FX-COVID**

Version no. 3.0 dated of 22/03/2021

The study will be carried out in accordance with the protocol, with current good practices and with statutory and regulatory requirements.

Coordinating Investigator
Dr. BRECHOT Nicolas
Intensive Care Unit
Hospital Pitié Salpêtrière
47-83 Boulevard de l'Hôpital – 75651 Paris Cedex 13

Date : ……………/………/………..
Signature :

Sponsor
Assistance Publique – Hôpitaux de Paris
Direction de la Recherche Clinique et de l'Innovation -
DRCI (Clinical Research and Innovation Department)
Hôpital Saint Louis
1 avenue Claude Vellefaux
75010 PARIS

Date : ……………/………/………..
Signature :

Collaborators

- **MChE Trading Handels-GmbH- F4-Pharma**
  Gersthofer Straße 103/23 1180, Wien, Austria
  Mail : thomas.steiner@mche.at
  Tel. + 43- 664-110 76 94

TABLE OF CONTENTS

1 SUMMARY 6

2 SCIENTIFIC JUSTIFICATION FOR THE STUDY 11

2.1 Hypothesis for the study 11

2.2 Description of knowledge relating to the condition in question 11

2.3 Summary of relevant pre-clinical experiments and clinical trials 11

2.4 Description of the population to be studied and justification for the choice of participants 11

2.5 Identification and description of the investigational medication or medications 12

2.6 Description and justification of the dosage, route of administration, administration schedule and treatment duration 12

2.7 Summary of the known and foreseeable benefits and risks for the research participants... 12

3 Objectives 13

3.1 Primary objective 13

3.3 Objective of any potential ancillary study 13

4 STUDY DESIGN 13

4.1 Study endpoints 13

4.2 Description of research methodology 14

5 Implementation of the study 17

5.1 Baseline visit = randomisation visit = D1 17

5.2 Follow-up visits 18

5.3 Day 30 21

5.4 Last study visit : Day 60 21

5.5 Expected length of participation and description of the chronology and duration of the study. 21

5.6 Table or diagram summarising the chronology of the study 21

5.7 Distinction between standard care and study 23

5.8 Biological samples collection 23

6 ELIGIBILITY CRITERIA 23

6.1 Inclusion criteria 23

6.2 Exclusion criteria 24

6.3 Recruitment procedure 24

6.4 Termination rules 24

7 TREATMENT ADMINISTERED TO STUDY PARTICIPANTS 26

7.1 Description of the investigational medicinal product(s) 26

7.2 Description of Additional medicinal product(s) (treatments required to conduct the study) 28

7.3 Description of traceability elements accompanying the investigational medicinal product(s) 28

7.4 Authorised and prohibited treatments (medicinal, additional medicinal, surgical), including rescue medications 28

7.5 Methods for monitoring compliance with the treatment 28

8 EFFICACY ASSESSMENT 29

8.1 Description of efficacy endpoints assessment parameters 29

8.2 Anticipated methods and timetable for measuring, collecting and analysing the efficacy data 29

9 Specific study committees 29

9.1 Steering Committee 29

10 SAFETY ASSESSMENT - RISKS AND BURDEN ADDED BY THE STUDY 29

10.1 Description of Safety endpoints assessment parameters 29

10.2 Anticipated methods and timetable for measuring, collecting and analysing the safety endpoints 29

10.3 Recording and reporting adverse events 29

11 data management 36

11.1 Data collection procedures 36
11.2 Identification of data recorded directly in the CRFs which will be considered as source data 36
11.3 Right to access data and source documents 36
11.4 Data processing and storage of research documents and data 37
11.5 Data ownership 37
12 statistical aspects 38
12.1 Description of statistical methods to be used including the timetable for the planned interim analyses 38
12.2 Calculation hypotheses for the number of participants required and the result 39
12.3 Anticipated level of statistical significance 39
12.4 Statistical criteria for termination of the study. 39
12.5 Method for taking into account missing, unused or invalid data 39
12.6 Management of modifications made to the analysis plan for the initial strategy. 39
13 Quality Control and ASSURANCE 39
13.1 General organisation 40
13.2 Quality control 40
13.3 Case report forms 40
13.4 Management of non-compliances 41
13.5 Audits/inspections 41
13.6 Principal Investigator’s commitment to assume responsibility 41
14 ETHICAL AND LEGAL CONSIDERATIONS 41
14.1 Methods for informing research participants and obtaining their consent 41
14.2 Prohibition from participating in another clinical study or exclusion period set after the study 42
14.3 Compensation for participants 42
14.4 Registration on the National Register of study participants to studies involving human participants concerning the products mentionned in Article L. 5311-1 of the Code de la santé publique 42
14.5 Authorisation for the research location 42
14.6 Legal obligations 42
15 Funding and Insurance 44
15.1 Funding sources 44
15.2 Insurance 44
16 Publication rules 44
16.1 Mention of AP-HP affiliation for projects sponsored by AP-HP 44
16.2 Mention of the sponsor AP-HP (DRCI) in the acknowledgements of the text 45
16.3 Mention of the financial backer in the acknowledgements of the text 45
17 Bibliography 46
18 List of addenda 47
18.1 List of investigators 47
18.2 Serious Adverse Events notification form 47
18.3 Pregnancy notification form 47
18.4 Investigator's Brochure 47
18.5 Immunogenicity – Final report 47

1 SUMMARY

**Full title**	FX06 to rescue acute respiratory distress syndrome during Covid-19 pneumonia
**Acronym/reference**	FX-COVID
**Coordinating investigator**	*Nicolas Bréchot, MD, PhD*<br>Medical-surgical ICU, La Pitié-Salpêtrière Hospital, Paris
**Sponsor**	Assistance Publique – Hôpitaux de Paris
**Scientific justification**	Vascular leakage following endothelial injury, responsible for interstitial and alveolar edema, is a major feature of pathogen induced acute lung injury [1,2]. As acute respiratory distress syndrome (ARDS) due to pandemic Covid-19 is associated with more than 60% mortality [3], controlling vascular leakage may be a major target to decrease the mortality associated with the spreading of the disease in France.<br>FX06, a drug under clinical development containing fibrin-derived peptide beta15-42, is able to stabilize cell-cell interactions, thereby reducing vascular leak and mortality in several animal models, particularly during lipopolysaccharide-induced and dengue hemorrhagic shock [4]. A phase I study was conducted in humans, with no specific adverse event detected with a dose up to 17.5 mg/kg. In a phase II randomized multicentre double-blinded trial in 234 patients suffering from ST+ acute coronary syndrome, FX06 treated patients exhibited a 58% decrease in the early necrotic core zone. Importantly, adverse events were highly comparable between groups, indicating a high safety profile for the drug [5]. Lastly, the drug was used as a salvage therapy in a patient exhibiting a severe ARDS following EBOLA virus infection [6]. Altogether, those data indicate that FX06 is well tolerated in humans and is a potent regulator of vascular leakage.<br>Our team will soon evaluate the efficacy of this drug in controlling vascular hyperpermeability during systemic inflammatory response syndrome following a cardiac arrest, a study founded by the PHRC (AOR19017). Our hypothesis here is that FX06 may decrease pulmonary vascular hyperpermeability during ARDS following SARS-CoV-2 infection, thereby improving gas exchanges and the outcome of infected patients.
**Main objective and primary endpoint**	The FX-Covid trial is a prospective, randomized, double-blinded, multicenter, controlled trial, testing the hypothesis that FX06 infusion in conjunction with optimal medical treatment during SARS-CoV-2 induced ARDS reduces pulmonary vascular hyperpermeability at 7 days, compared to optimal medical treatment alone.<br><br>**The primary endpoint will be the change in extravascular lung water index (EVLWi) assessed by transpulmonary thermodilution between Day 1 and Day 7.**<br>Transpulmonary thermodilution systems, part of the standard management in ICU, allow a direct evaluation of vascular hyperpermeability in the lungs using thermodilution technique. EVLWi is a reliable parameter,

	independently associated with mortality during ARDS [2].
**Secondary objectives and endpoints**	The trial will test also the hypothesis that FX-06 infusion:<br>- improves mortality at 30 days following randomization<br>- reduces vascular leak (weight, fluid balance, systolic, diastolic, mean blood pressure, heart rate, catecholamine and lactate clearance)<br>- improves pulmonary recovery<br>- improves the evolution of organ failure<br>- reduces the need for hemodynamic support with catecholamines<br>- reduces the need for renal dialysis<br><br>The study will also evaluate the tolerance of FX06, its pharmacokinetic (PK/PD analysis) and immunogenicity.<br><br>**Accordingly, secondary endpoints will be:**<br><br>• Evolution of daily extravascular lung water index (EVLWi), cardiac index, global end-diastolic volume index, pulmonary vascular permeability index measured by transpulmonary thermodilution during 7 days.<br>• Overall survival at 30 days<br>• Mortality rate in ICU, in hospital<br>• Rate of withdraw or withhold life-sustaining treatments decision at Day 30<br>• Vascular leak:<br>o Daily weight until D7 (% of D1)<br>o Daily fluid balance until D7<br>o Evolution of albuminemia until D7<br>• Pulmonary recovery:<br>o Duration of mechanical ventilation (MV) and MV free days at D30<br>o Ventilator-free survival (proportion of participants alive and off invasive mechanical ventilation) at D30<br>o PaO_2/FiO_2 and Murray ARDS severity score over 15 days<br>o Evolution of pulmonary SOFA score over 15 days<br>o Evolution of radiological Weinberg score over 30 days, analysed by a specialized radiologist, blinded for the treatment group<br>o Rate of rescue therapy with VV-ECMO<br>• Evolution of sequential organ failure score (SOFA) and organ failure (one or more SOFA sub-score >=3) failure free days at D15<br>• Hemodynamic parameters<br>o systolic, diastolic, mean blood pressure, heart rate during 7 days<br>o lactate clearance, measured once a day during 7 days<br>o catecholamine-free days at D30<br>• Duration of renal replacement therapy (RRT), and

	RRT free days at D30<br>• Nature and frequency of adverse events<br>• Evolution of FX06 concentration from H0 to H1, measured at time 0 (before FX06 application) and after 5, 15, 30, 60 min<br>• PK/PD analysis will be performed from those dosages, with a reduction of EVLWi of more than 30% before and 3 hours after injection as primary PD endpoint at day 2<br>• Immunogenicity of the drug will be tested at day 7, according to manufacturer's procedure (cf 5.7)<br>Exploratory objectives will be:<br>- To characterize responder patients (defined as patient with a change in extravascular lung water index between D1 and Day 7 after inclusion > 30%)<br>- To study the prognosis (overall mortality) of responder patients (defined as patient with a change in extravascular lung water index between D1 and Day 7 after inclusion > 30%).
**Design of the study**	Comparative, double-blinded, controlled (1:1), randomized trial, assessing the effect of FX06 infusion on the top of standard of care *vs* standard of care alone in 2 parallel arms, and multicentric (3 ICUs from three different hospitals).
**Population of study participants**	Patients admitted in participating ICUs who received mechanical ventilation for SARS-CoV-2 induced acute respiratory distress syndrome (ARDS)
**Inclusion criteria**	1. Age ≥ 18 years<br>2. SARS-CoV-2 induced pneumonia confirmed by a positive PCR test in nasopharyngeal swab or respiratory tract secretions<br>3. Acute respiratory distress syndrome (ARDS) according to Berlin criteria (bilateral pulmonary infiltrates on frontal chest x-ray, PaO2/FiO2 ratio ≤300 mmHg, objective assessment excluding hydrostatic pulmonary edema)<br>4. Need for endotracheal intubation and mechanical ventilation<br>5. Informed consent by patient or legal representative. According to the specifications of emergency consent, randomization without the close relative or surrogate consent could be performed.<br>6. Affiliated to a social security system<br>7. Highly effective method of contraception and negative highly sensitive pregnancy test, for women of childbearing potential
**Exclusion criteria**	1. Mechanically ventilation for more than 4 days<br>2. Patient receiving drugs interfering with inflammation: non-steroidal anti-inflammatory drugs, immunoglobulins.<br>3. Patients receiving chemotherapy, radiotherapy or immunotherapy for malignancy<br>4. Participation in another interventional clinical trial<br>5. Pregnant or lactating women<br>6. Patient moribund on the day of randomization, defined by a SAPS-II score>90

	7. Contra-indication for vascular access implantation for transpulmonary thermodilution monitoring<br>8. Severe or terminal renal insufficiency (creatinine clearance <30 ml/min)<br>9. Severe hepatic insufficiency (hepatic SOFA score>2)<br>10. Severe cardiac insufficiency, with left ventricular ejection fraction<30%<br>11. Any history of severe allergic drug reaction (anaphylactic shock or allergic angioedema)<br>12. Persons deprived of their liberty by a judicial or administrative decision (guardianship or tutelage measure)
**Investigational medicinal product(s)**	Development phase IIa<br>FX06 i.v.: 400 mg per day (divided in two injections) during 5 days
**Comparator treatment**	Equivalent placebo
**Interventions added for the study**	None other than treatment or placebo injection. Particularly, EVLWI monitoring is part of the routine care of ARDS patients.<br>Chest X-Rays (D15 and D30, only if hospitalized)<br>Phone call at D30 and D60 in case of discharge from the ICU<br>Biological samples for pharmacokinetic study, as well as immunogenicity testing and IL6 dosage
**Expected benefits for the participants and for society**	Considering the criteria for inclusion, the population included in the trial has a predicted mortality around 60% [3], with severity of vascular leakage associated with mortality [2]. FX06 was able to attenuate vascular leakage in several animal models [4]. It was responsible for a 58% decrease in the MRI evaluated early necrotic zone in a phase II randomized multicentre double-blinded trial conducted in 234 patients suffering from ST+ acute coronary syndrome, without any detectable side effect [5], and successfully rescued a patient with EBOLA-induced ARDS [6]. We therefore believe that the benefit/risk ratio strongly supports the administration of the drug in the study population.<br>More generally, if positive, it will provide a new treatment to decrease the mortality associated with the spreading of SARS-CoV-2 disease worldwide.
**Risks and burdens added by the study**	Risks due to the treatments<br>D level
**Number of participants included**	50
**Number of centres**	5 ICUs in three different hospitals, national setting
**Duration of the study**	- inclusion period: 7 months<br>- participation period (treatment + follow-up): 60days<br>- total duration: 9 months
**Number of enrolments expected per site and per month**	1,4
**Statistical analysis**	Analysis will be made with an intent-to-treat design, without interim analysis.<br>The primary endpoint, change of EVLWI from D1 to D7, will be compared between the two groups using analysis of covariance (ANCOVA).<br>For patients dying before 7 days, the last thermodilution data will be retained for the primary analyses. If the patient's

	condition no longer warrants the presence of the transpulmonary thermodilution systems, then the catheter is removed before 7 days. In this case, the measurement of EVLWi can no longer be made, the last thermodilution data will be retained for the primary analyses. A sensitivity excluding those patients will be performed for analysing the primary endpoint.
**Funding sources**	- MChE Handelsges m.b.H (F4-Pharma), Vienna, Austria, will provide the drug, and participates in the funding of the study<br>- Bouygues SA<br>- Sanofi SA<br>None of the investigators has any conflict of interest with this study.
**Study will have a Data Safety Monitoring Board**	Yes

2 SCIENTIFIC JUSTIFICATION FOR THE STUDY

2.1 Hypothesis for the study

The main hypothesis of FX-Covid trial is that FX-06 may attenuate vascular leakage during SARS-CoV-2 induced acute respiratory distress syndrome (ARDS), with beneficial effects on the outcomes of the patients.

2.2 Description of knowledge relating to the condition in question

Vascular leakage is a major feature of pathogen induced acute lung injury [1,2]. It is triggered by high levels of inflammation in the lungs, due to pathogen-induced endothelial and epithelial lesions. Accordingly extravascular lung water index (EVLWI), a physiological marker of pulmonary vascular leak measured by transpulmonary thermodilution, is independently associated with the outcome of the patients during ARDS [2,7,8]. As ARDS due to COVID-19 infection is associated with more than 60% mortality [3], controlling vascular leakage may be a major target to decrease the mortality associated with the spreading of the disease.

2.3 Summary of relevant pre-clinical experiments and clinical trials

FX-06, a drug arising from research on fibrin-derived peptide Bβ15-42, is able to stabilize VE-cadherin dependent cell-cell interactions, thereby reducing vascular leak [9,10]. It was shown to reduce capillary leakage *in vitro* [4] and in several animal models [4,11–13]. It significantly decreased LPS induced pulmonary leakage in rats, and offered a protection from mortality in a model of dengue-virus infection in mice [4].

A randomized placebo-controlled phase I study was conducted in humans, in which no specific adverse event could be detected with a dose escalation up to 17.5 mg/kg [12]. A phase II randomized multicentre double-blinded trial was then conducted in 234 patients suffering from ST+ acute coronary syndrome. FX-06 was injected as a single 400 mg dose at reperfusion. The primary end-point, which was the infarcted size at day 5 evaluated by late gadolinium enhancement during cardiac MRI, was not statistically different between groups, but patients FX-06 treated exhibited a 58% decrease in the early necrotic core zone, indicating myocardial edema [5]. Importantly, adverse events were highly comparable between groups, indicating a high safety profile for the drug. Lastly, the drug was used with success as a salvage therapy in a patient exhibiting a severe ARDS following EBOLA virus infection [6]. More recently FX06 was used as compassionate therapy in 3 university centers in Germany, in 6 highly severe patients receiving extra-corporeal membrane oxygenation for SARS-CoV-2 disease. The dosage consisted in 400/day during 4 to 7 days. Four over six patients could improve their condition, while 2 died. No clear adverse event related to the treatment could be detected in this cohort (data submitted for publication).

Altogether, those data indicate that FX-06 is well tolerated in humans and is a potent regulator of vascular leakage during ARDS. Our hypothesis here is that FX06 may decrease pulmonary vascular hyperpermeability during ARDS following SARS-CoV-2 infection, thereby improving gas exchanges and the outcome of infected patients.

2.4 Description of the population to be studied and justification for the choice of participants

The population studied will be all patients admitted in one of the participating ICUs exhibiting SARS-CoV-2 induced ARDS and receiving mechanical ventilation.

Justification:

- The mortality of those severe forms of the disease is very high, around 60% [3], justifying an urgent research to find an appropriate treatment.
- The level of vascular leak is an independent predictor of mortality during ARDS.

2.5 Identification and description of the investigational medication or medications

FX06, developed by F4-Pharma GmbH, Vienna, Austria, is an injectable drug arising from research on fibrin-derived peptide Bβ15-42. It is able to stabilize VE-cadherin dependent cell-cell interactions, thereby reducing vascular leak.
Concerning tolerance, no specific adverse event could be detected with a dose escalation up to 17.5 mg/kg in a randomized placebo-controlled phase I study in humans [12]. Adverse events were also highly comparable between groups in a phase II randomized multicentre double-blinded trial in patients with ST+ acute coronary artery syndrome [5]. Altogether, these data indicate a high safety profile for the drug.
The dosage will consist in a daily dose of 400 mg/day during 5 days, divided in two injections per day [6].

2.6 Description and justification of the dosage, route of administration, administration schedule and treatment duration

In two other pig models of hemorrhagic shock induced SIRS, one of them with blinding for allocation group, FX06 was responsible for an increased cardiac index and less pulmonary lesions, as indicated by an increase in PaO2/FiO2 ratio and a decrease in extra-vascular lung water. FX06 group exhibited also an almost complete abolishment of circulating endotoxemia [13,14]. Doses used in these studies were a single bolus of 2.4 mg/kg at reperfusion [14], and a bolus of 3 mg/kg followed by a continuous infusion of 18 mg/kg/h during 1 hour [13].
In two randomized placebo-controlled phase I studies conducted in humans, no specific adverse event could be detected with a dose escalation up to 17.5 mg/kg [12] , and [internal report: *Study FX06AQ-101 [report no. CRO-05-68], First dose in man of FX06, a new peptide preventing inflammation and damage of post-infarction myocardial tissue*]. A phase II randomized multicentre double-blinded trial was then conducted in 234 patients suffering from ST+ acute coronary syndrome. Fx-06 was injected as a single 400 mg dose at reperfusion. The primary end-point, which was the infarcted size at day 5 evaluated by late gadolinium enhancement during cardiac MRI, was not statistically different between groups, but patients FX-06 treated exhibited a 58% decrease in the early necrotic core zone [5]. Importantly, adverse events were highly comparable between groups, indicating a high safety profile for the drug. Lastly, the drug was used as a salvage therapy in a patient exhibiting a severe ARDS following EBOLA virus infection, with a favourable issue. Treatment schedule was a bolus of 400 mg followed by a dose of 200 mg every 12 hrs during 3 days [6]. More recently FX06 was used as compassionate therapy in 3 university centers in Germany, in 6 highly severe patients receiving extra-corporeal membrane oxygenation for SARS-CoV-2 disease. The dosage consisted in 400/day during 4 to 7 days. Four over six patients could improve their condition, while 2 died. No clear adverse event related to the treatment could be detected in this cohort (data submitted for publication). We therefore retained the dosage of 400 mg per day, divided in two injections, and during 5 days, for our study.

2.7 Summary of the known and foreseeable benefits and risks for the research participants

2.7.1 Individual benefit/risk ratio

Considering the criteria for inclusion, the population included in the trial has a predicted mortality around 60% [3], with severity of vascular leakage associated with mortality [2]. FX-06 was able to attenuate vascular leakage in several animal models [4]. It was responsible for a 58% decrease in the MRI evaluated early necrotic zone in a phase II randomized multicentre double-blinded trial conducted in 234 patients suffering from ST+ acute coronary syndrome, without any detectable side effect [5], and successfully rescued a patient with EBOLA-induced ARDS [6]. We therefore believe that the benefit/risk ratio strongly supports the administration of the drug in the study population.

2.7.2 Constraints imposed by the research

Constraints for patients included will be limited (for more details see the section 4, "description of the trial"):

- Administration of the study drug will be made with an IV route, at a time the patients are sedated and equipped routinely with a central venous catheter.
- Recording of clinical parameters and outcome will not differ from usual practice. Particularly, Evaluation of EVLW and other thermodilution parameters using transpulmonary thermodilution technique is part of the standard of care during ARDS [15].
- Biological samples for pharmacokinetic study, as well as immunogenicity testing and IL6 dosage (see 5.7)

3 OBJECTIVES

3.1 Primary objective

The FX-Covid trial is a prospective, randomized, double-blinded, multicenter, controlled trial, testing the hypothesis that FX06 infusion in conjunction with optimal medical treatment during SARS-CoV-2 induced ARDS reduces pulmonary vascular hyperpermeability at 7 days, compared to optimal medical treatment alone.

3.2 Secondary objectives

Secondary objectives will be to test the hypothesis that FX06 infusion:

- improves mortality at 30 days following randomization
- reduces capillary leak (weight, fluid balance, systolic, diastolic, mean blood pressure, heart rate, catecholamine and lactate clearance)
- improves pulmonary recovery
- improves the evolution of organ failure
- reduces the need for hemodynamic support with catecholamines
- reduces the need for renal dialysis

The study will also determine

- the tolerance of FX06
- its pharmacokinetic
- PK/PD parameters of the drug
- Immunogenicity induced by the drug

Exploratory objectives will be:

- To characterize responder patients (defined as patient with a change in extravascular lung water index between D1 and Day 7 after inclusion > 30%)
- To study the prognosis (overall mortality) of responder patients (defined as patient with a change in extravascular lung water index between D1 and Day 7 after inclusion > 30%).

3.3 *Objective of any potential ancillary study*

Not applicable

4 STUDY DESIGN

4.1 *Study endpoints*

4.1.1 Primary endpoint

Change in extravascular lung water index (EVLWi) assessed by transpulmonary thermodilution between D1 and Day 7 after inclusion.

Transpulmonary thermodilution systems, part of the standard management in ICU, allow a direct evaluation of vascular hyperpermeability in the lungs. Extra-vascular lung water index (EVLWi) is a reliable parameter, independently associated with mortality during ARDS [2].

4.1.2 Secondary endpoints

- Evolution of daily extravascular lung water index (EVLWi), cardiac index, global end-diastolic volume index, pulmonary vascular permeability index, measured by transpulmonary thermodilution during 7 days.
- Overall survival at 30 days
- Mortality rate in ICU, in hospital
- Rate of withdraw or withhold life-sustaining treatments decision at Day 30
- Vascular leak:
    - Daily weight until D7 (% of D1)
    - Daily fluid balance until D7
    - Evolution of albuminemia until D7
- Pulmonary recovery:
    - Duration of mechanical ventilation (MV) and MV free days at D30
    - Ventilator-free survival (proportion of participants alive and off invasive mechanical ventilation) at D30
    - PaO_2/FiO_2 and Murray ARDS severity score over 15 days
    - Evolution of pulmonary SOFA score over 15 days
    - Evolution of radiological Weinberg score over 30 days, analysed by a specialized radiologist, blinded for the treatment group
    - Rate of rescue therapy with VV-ECMO
- Evolution of daily sequential organ failure score (SOFA) and organ failure (one or more SOFA sub-score ≥3) failure free days at D15
- Hemodynamic parameters
    - systolic, diastolic, mean blood pressure, heart rate twice a day during 7 days
    - lactate clearance, measured once a day during 7 days
    - catecholamine-free days at D30
- Duration of renal replacement therapy (RRT), and RRT free days at D30
- Nature and frequency of adverse events
- Evolution of FX06 concentration from H0 to H1, measured at time 0 (before FX06 application) and after 5, 15, 30, 60 min
- PK/PD analysis will be performed from those dosages, with a reduction of EVLWi of more than 30% before and 3 hours after injection as primary PD endpoint at day 2
- Immunogenicity of the drug will be tested at day 7, according to manufacturer’s procedure (cf 5.7)

4.2 *Description of research methodology*

4.2.1 Design of the study

Comparative, double-blinded, controlled (1:1), randomized trial, assessing the effect of FX06 infusion on the top of standard of care *vs* standard of care alone in 2 parallel arms, and multicentric (3 ICUs from three different hospitals).

4.2.2 Participating sites

Different ICUs from different hospitals.

- **Recruitment centres**

Intensive care units

Numéros centres	Hôpital/Adresse	Service	Investigateur
**001**	Groupe Hospitalier Pitié Salpêtrière	Service de Médecine Intensive Réanimation du Pr. Alain COMBES	Dr BRECHOT Nicolas
**002**	Centre Hospitalier Universitaire d´Anger	Service de Médecine Intensive Réanimation du Pr. Alain MERCAT	Dr ASFAR Pierre
**003**	Centre Hospitalier Intercommunal de Poissy-Saint Germain en Laye, site de Poissy	Service de Médecine Intensive Réanimation du Pr Outin	Pr AYON Jan
**004**	Groupe Hospitalier Pitié Salpêtrière	Service de Médecine Intensive Réanimation Neurologique	Dr LE GUENNEC Loïc
**005**	Centre Hospitalier Universitaire d´Anger	Service d'Anesthésie - Réanimation chirurgicale	Pr LASOCKI Sigismond

- **Non-recruitment centres**

None

4.2.3 Identification of participants

The subjects participating in this study will be identified as follows:
Site number (3 digits) - Sequential selection number for the site (4 digits) - surname initial - first name initial
This reference number is unique and will be used for the entire duration of the trial.

A randomisation number will also be assigned when the participant is randomised. This number will have the following format: XXX.
A treatment number, formatted as T0000 (1 letter and 4 numbers), will be also assigned when the participant is allocated to a treatment arm.

4.2.4 Randomisation

The randomization list will be computer-generated and stratified according to each ICU.
Patients will be randomly assigned in a 1:1 ratio to FX06 or standard of care.
Concealment of the study-group assignments is achieved with the use of a centralized, secure, interactive, Web-based response system (CleanWeb, Telemedicine Technologies) accessible from each study center.

After each randomization, a treatment number is allocated to the patient and a confirmation email of randomization is sent automatically to the investigator concerned and the URC.

4.2.5 Blinding methods and measures put in place to protect blinding

This study will be conducted in a blinded fashion. To maintain blinding of study treatment, unrecognizable study medications (numbered and sealed Therapeutic Units = boxes containing 10 vials of active or placebo solution) will be prepared.

Subjects, Investigators and the Sponsor's staff conducting the study, will not have access to individual subject treatment assignments. The Randomization Center will have access to such assignments. Blinding is critical to the integrity of this clinical study. However, in the event of a medical emergency or pregnancy in an individual subject, in which knowledge of the investigational product is critical to the subject's management, the blind for that subject may be broken by the treating investigator.

Before breaking the blinding of an individual subject's treatment, the investigator should have determined that the information is necessary, i.e. that it will alter the subject's immediate management. In many cases, particularly when the emergency is clearly not investigational product-related, the problem may be properly managed by assuming that the subject is receiving active product without the need for unblinding.

4.2.6 Unblinding procedures

Unblinding will be requested for any reason considered essential by the investigating doctor by calling upon:

- **In emergency cases** at the poison control center at Fernand Widal Hospital, Telephone: **+33 (0)1 40 05 48 48.** In the case of unblinding the Sponsor will be informed immediately.
- **Apart from an emergency situation** at the CRID with the CRID project advisor whose contact information is listed on the protocol cover page. The request should be addressed on working days and during working hours.

5 IMPLEMENTATION OF THE STUDY

5.1 Baseline visit = randomisation visit = D1

Informed consent will be obtained by investigator of each center from a close relative or surrogate. When such a person will be absent, the patient will be randomized according to French law specifications for emergency consent and patient's surrogate will be asked to give his or her consent for continuation of the trial, and the patient's himself when his or her condition will allow it permitted.

Inclusion and exclusion criteria will be verified by each investigator before inclusion, using a web based questionnaire, blood pregnancy test will be performed (if applicable) and the patient will be randomized through a web-based procedure.

**Whose consent must be obtained**	**Who informs the individual and collects their consent**	**When is the individual informed**	**When is the individual's consent collected**
• *Patient's legal representative, family member or a close relative*	• *Local site investigator*	• *For patient's legal representative, family member or close relative : Before randomization OR as soon as possible after randomisation*<br>• *For patient: When his/her condition will allow*	• *For patient's legal representative, family member or close relative : Before randomization OR as soon as possible after randomisation*<br>• *For patient: When his/her condition will allow*

- Demographic and usual clinical data will be extracted from medical charts.
  - Date of inclusion in the FX-COVID study
  - Demographic data: Age, sex, Weight and size, Charlson and Mc Cabe scores, previous medical condition (Diabetes, ischemic cardiomyopathy, cardiac insufficiency, renal insufficiency, chronic dialysis, chronic respiratory insufficiency, COPD, liver cirrhosis, cancer, Immunocompromized, other)
  - Symptoms duration before inclusion
  - Specific treatment received for SARS-CoV-2 before inclusion (CTC, Remdesivir)
  - Diuretics
  - Duration of mechanical ventilation before inclusion
  - Co-infection or not
  - Weight of the patient
  - Ongoing prone-positioning (prone positioning on the day on the evaluation)
  - Standard clinical monitoring (arterial pressure, heart rate)
  - Ventilatory settings :
    - VV-ECMO Y/N
    - Ventilatory mode (ACV, PSV, APRV, spontaneous breathing)
    - Tidal volume
    - Respiratory rate
    - Positive end-expiratory pressure (PEEP)
    - Plateau pressure
    - FiO_2
  - Level of catecholamines: inotrope score, calculated as IS= dose of dubutamine (µg/kg/min)+ 100 x [dose of norepinephrine (µg/kg/min) + dose of epinephrine (µg/kg/min)] [16]
  - Supplementation by hydrocortisone Y/N
  - Biological exams :

    - Arterial blood gases
    - Haematology (blood count)
    - Ionogram, renal and hepatic markers
    - serum glucose, CRP, LDH, NT-pro-BNP, procalcitonin, troponin
    - Coagulation tests: PT ratio, aPTT, Fibrinogenemia, D-Dimers
    - Albuminemia
  - ECG
  - SOFA score
  - RRT or not
  - Chest x-ray
  - Trans-thoracic echocardiography

- Assessment of transpulmonary thermodilution (TPT) parameters. **One measurements will be made before administration of the treatment [7]. All parameters will be evaluated in supine position. Parameters recorded will be: Extra-vascular lung water index, Global end-diastolic volume index, pulmonary vascular permeability index, and cardiac index.** Central venous and arterial lines used for the routine care of the patient will be compatible with TPT systems (either PICCO, Getinge, Germany or EV1000, Edwards Lifesciences, US).

- Treatment
  - Administration by IV perfusion 400mg FX06 intravenously or placebo as a two-time bolus within 10 minutes interval)
  - Administration as soon as possible after randomization, but no longer than 12 hours.

- Pharmacokinetic study: Additional 1 ml blood sample will be taken at time 0 (before FX06 application) and after 5, 15, 30, 60 min. Because FX06 in blood is quickly degraded by endopeptidases, the following sampling procedure should be followed: 1 ml blood to be collected into tubes containing Li-heparin, mixed by inversion 10 times, and then 0.8 ml of this blood to be transferred to test tubes containing 0.08 ml of 0.88 M HCl. It is critical that blood is being transferred to acid within 2 min of collection. The tubes are closed, the acidified blood samples will be mixed by inversion 10 times, and then processed to plasma by centrifugation (2500g; 10 min). Acidified plasma samples will be stored at a temperature of less than -20°C. FX06 dosage will be processed according by F4-Pharma, according to its dedicated procedure. Alternatively, pharmacokinetic study may be performed every other day of treatment (D1 to D5).

5.2 Follow-up visits

5.2.1 D2 to D5 (all data recorded at 10 O'Clock in the morning)

  - Vital status
  - Weight of the patient
  - Daily fluid balance
  - Co-infection or not
  - Specific treatment received for SARS-CoV-2 (CTC, Remdesivir)
  - Diuretics
  - RRT or not
  - Ongoing prone-positioning (prone positioning on the day on the evaluation)
  - Standard clinical monitoring (arterial pressure, heart rate)
  - Ventilatory settings:
    - VV-ECMO Y/N
    - Ventilatory mode (ACV, PSV, APRV, spontaneous breathing)
    - Tidal volume
    - Respiratory rate
    - Positive end-expiratory pressure (PEEP)
    - Plateau pressure
    - FiO_2
  - Level of catecholamines: inotrope score, calculated as IS= dose of dobutamine (µg/kg/min)+

100 x [dose of norepinephrine (µg/kg/min) + dose of epinephrine (µg/kg/min)] [16]
- Supplementation by hydrocortisone Y/N
- Biological exams:
  - Arterial blood gases
  - Haematology (blood count)
  - Ionogram, renal and hepatic markers
  - serum glucose, CRP, LDH, NT-pro-BNP, procalcitonin, troponin
  - Coagulation tests: PT ratio, aPTT, fibrinogenemia, D-Dimers
  - Albuminemia
- ECG
- Use of renal replacement therapy
- SOFA score
- Chest x-ray
- Trans-thoracic echocardiography

- Assessment of transpulmonary thermodilution (TPT) parameters. **Two measurements will be made, one before administration of the treatment, and one 3 hours after at day 2 [7]**. For day 3 to D5, one measurement will be made before administration of the treatment. **All parameters will be evaluated in supine position. Parameters recorded will be: Extra-vascular lung water index, Global end-diastolic volume index, pulmonary vascular permeability index, and cardiac index.** Central venous and arterial lines used for the routine care of the patient will be compatible with TPT systems (either PICCO, Getinge, Germany or EV1000, Edwards Lifesciences, US).

- Treatment
  - Administration by IV perfusion (400mg FX06 intravenously or placebo as a two-time bolus within 10 minutes interval)

5.2.2 D6 to D7 (all data recorded at 10 O'Clock in the morning)

- Vital status
- Weight of the patient
- Daily fluid balance
- Co-infection or not
- Specific treatment received for SARS-CoV-2 (CTC, Remdesivir)
- Diuretics
- RRT or not
- Ongoing prone-positioning (prone positioning on the day on the evaluation)
- Standard clinical monitoring (arterial pressure, heart rate)
- Ventilatory settings:
  - VV-ECMO Y/N
  - Ventilatory mode (ACV, PSV, APRV, spontaneous breathing)
  - Tidal volume
  - Respiratory rate
  - Positive end-expiratory pressure (PEEP)
  - Plateau pressure
  - FiO_2
- Level of catecholamines: inotrope score, calculated as IS= dose of dobutamine (µg/kg/min)+ 100 x [dose of norepinephrine (µg/kg/min) + dose of epinephrine (µg/kg/min)] [16]
- Supplementation by hydrocortisone Y/N
- Biological exams:
  - Arterial blood gases
  - Haematology (blood count)
  - Ionogram, renal and hepatic markers
  - serum glucose, CRP, LDH, NT-pro-BNP, procalcitonin, troponin
  - Coagulation tests: PT ratio, aPTT, fibrinogenemia, D-Dimers

  - Albuminemia
- ECG
- Use of renal replacement therapy
- SOFA score
- Chest x-ray
- Trans-thoracic echocardiography at D7
- Serum sample for immunogenicity and IL6 measurement at D7

- Assessment of transpulmonary thermodilution (TPT) parameters. **One measurement per day will be made [7]**. **All parameters will be evaluated in supine position. Parameters recorded will be: Extra-vascular lung water index, Global end-diastolic volume index, pulmonary vascular permeability index, and cardiac index.** Central venous and arterial lines used for the routine care of the patient will be compatible with TPT systems (either PICCO, Getinge, Germany or EV1000, Edwards Lifesciences, US).

5.2.3 D8, D10, D15

- Vital status
- Specific treatment received for SARS-CoV-2 (CTC, Remdesivir)
- Diuretics
- Ventilatory settings:
  - Ventilatory mode (ACV, PSV, APRV, spontaneous breathing)
  - Tidal volume
  - Respiratory rate
  - Positive end-expiratory pressure (PEEP)
  - Plateau pressure
  - FiO_2
- Level of catecholamines: inotrope score, calculated as IS= dose of dobutamine (µg/kg/min)+ 100 x [dose of norepinephrine (µg/kg/min) + dose of epinephrine (µg/kg/min)] [16]
- Biological exams:
  - Arterial blood gases (if clinically indicated)
- Use of renal replacement therapy
- SOFA score
- Chest x-ray (at D15 if clinically indicated)

5.2.4 Date of ICU discharge or death

- Vital status
- Specific treatment received for SARS-CoV-2 (CTC, Remdesivir)
- Diuretics
- Ventilatory settings:
  - Ventilatory mode (ACV, PSV, APRV, spontaneous breathing)
  - Tidal volume
  - Respiratory rate
  - Positive end-expiratory pressure (PEEP)
  - Plateau pressure
  - FiO_2
- Level of catecholamines: inotrope score, calculated as IS= dose of dobutamine (µg/kg/min)+ 100 x [dose of norepinephrine (µg/kg/min) + dose of epinephrine (µg/kg/min)] [16]
- Biological exams:
  - Arterial blood gases
- Use of renal replacement therapy
- Chest X-ray if hospitalized and clinically indicated
- SOFA score
- Prescription of a highly efficient method of contraception (oral contraception associated with

inhibition of ovulation, intrauterine device or hormone releasing system, bilateral tubal occlusion, vasectomized partner or sexual abstinence for more than three months after inclusion), for ICU discharge of women for childbearing potential

5.3 Day 30

- Vital status
- Chest X-ray if hospitalized and clinically indicated
- Rate of withdraw or withhold life-sustaining treatments decision at Day 30
- Duration of mechanical ventilation (MV) and MV free days at D30
- Ventilator-free survival (proportion of participants alive and off invasive mechanical ventilation) at D30
- Secondary ventilator-associated pneumonia, nb of episodes
- Rescue therapy with VV-ECMO
- Catecholamine-free days at D30
- Duration of renal replacement therapy (RRT), and RRT free days at D30
- High sensitive pregnancy test

5.4 Last study visit : Day 60

An additional visit at D60 will be made by investigators by phone, asking to the medical staff in charge of the patient for the patient's status (dead, hospitalized with high-flow oxygen (more than 5 L/min), hospitalized with low-flow oxygen therapy, discharged from hospital). Serious adverse events will be also collected at this time point.

5.5 Expected length of participation and description of the chronology and duration of the study.

Length of Inclusion period	7 months
Duration of participation for each subject, of which:	
• Treatment period:	5 days
• Follow-up period with treatment period:	60 days
Total study duration:	9 months

5.6 Table or diagram summarising the chronology of the study

Actions	D1	D2 to D5	D6 to D7	D8, D10, D15, ICU discharge, death	D30	D60
Information	X					
Signature of the consent form from a close relative or surrogate, or emergency consent form	X					
Randomization	X					
Administration of study drug or placebo	X	X				
Pharmacokinetic study	X					
Medical history	X					
Clinical exam	X					
Co-infection or not	X	X	X			

Weight	X	X	X			
Fluid balance		X	X			
EVLWI measured by TPT (primary endpoint)		X	X			
Standard clinical monitoring	X	X	X			
Ventilatory settings	X	X	X	X		
Doses of catecholamines	X	X	X	X		
Arterial blood gases	X	X	X	X*		
Blood pregnancy test	X				X	
NFS, TP, TCA, fibrinogen, D-Dimers	X	X	X			
Electrolytes, creatininemia, serum glucose, CRP, LDH, NT-pro-BNP, procalcitonin, troponin	X	X	X			
ECG	X	X	X			
Liver tests	X	X	X			
Albuminemia	X	X	X			
SOFA score	X	X	X	X		
RRT or not	X	X	X	X	X	
TTE	X	X	X (D7)			
Chest X-RAY	X	X	X	X*	X*	
Adverse events		X	X	X	X	
Pursuit consent		X	X	X	X	
Vital status					X	
Withhold life-sustaining treatments decision					X	
Ventilator-free survival					X	
Secondary pneumonia, nb of episodes					X	
Rescue therapy with VV-ECMO					X	
Catecholamine-free days at D30					X	
RRT free days at D30					X	
Serum sample for immunogenicity and IL6 measurement			X (D7)			
Prescription of a highly effective method of contraception for three months (for women of childbearing potential)				X (ICU discharge)		
Patient’s status						X

*, if clinically indicated

5.7 Distinction between standard care and study

Procedures and treatments carried out as part of the research	Procedures and treatments associated with care	Procedures and treatments added because of the research
**Treatments**	Standard of care	FX-06 administration or its placebo at D1, D2, D3, D4, D5
**Consultations**	D1 to D30: During patient's hospitalisation's	Follow up by phone: at D30 and D60 in case of discharge from the ICU
**Blood samples**	Haematology - Biochemistry	Day 1 : 1 ml at time 0 (before FX06 application) and after 5, 15, 30, 60 min<br>Day 7 : 3 ml serum
**Imaging, etc.**	TTE<br>Chest X-Rays<br>ECG	None
**Monitoring**	Transpulmonary thermodilution technique	

Particularly, transpulmonary thermodilution technique is part of the routine monitoring during ARDS [15].

5.8 Biological samples collection

- Pharmacokinetic study: Additional 1 ml blood sample will be taken at time 0 (before FX06 application) and after 5, 15, 30, 60 min. Because FX06 in blood is quickly degraded by endopeptidases, the following sampling procedure should be followed: 1 ml blood to be collected into tubes containing Li-heparin, mixed by inversion 10 times, and then 0.8 ml of this blood to be transferred to test tubes containing 0.08 ml of 0.88 M HCl. It is critical that blood is being transferred to acid within 2 min of collection. The tubes are closed, the acidified blood samples will be mixed by inversion 10 times, and then processed to plasma by centrifugation (2500g; 10 min). Acidified plasma samples will be stored at a temperature of less than -20°C. FX06 dosage will be processed by F4-Pharma, according to its dedicated procedure.

- An additional 3 ml serum collection will be performed at day 7, and frozen for further immunogenicity tests by the manufacturer (addendum 18.5), as well as IL6 quantification.

6 ELIGIBILITY CRITERIA

6.1 Inclusion criteria

1. Age ≥ 18 years
2. SARS-CoV-2 induced pneumonia confirmed by a positive PCR test in nasopharyngeal swab or respiratory tract secretions
3. Acute respiratory distress syndrome (ARDS) according to Berlin criteria (bilateral pulmonary infiltrates on frontal chest x-ray, PaO2/FiO2 ratio ≤300 mmHg, objective assessment excluding hydrostatic pulmonary oedema)
4. Need for endotracheal intubation and mechanical ventilation
5. Informed consent by patient or legal representative According to the specifications of emergency consent, randomization without the close relative or surrogate consent could be performed.
6. Affiliated to a social security system

7. Highly effective method of contraception (oral contraception associated with inhibition of ovulation, intrauterine device or hormone releasing system, bilateral tubal occlusion, vasectomized partner or sexual abstinence for more than three months before inclusion) and negative highly sensitive pregnancy test, for women of childbearing potential

6.2 Exclusion criteria

1. Mechanically ventilation for more than 4 days
2. Patient receiving drugs interfering with inflammation: non-steroidal anti-inflammatory drugs, immunoglobulins.
3. Patients receiving chemotherapy, radiotherapy or immunotherapy for malignancy
4. Participation in another interventional clinical trial
5. Women pregnant or lactating
6. Patient moribund on the day of randomization, defined by a SAPS-II score>90
7. Contra-indication for vascular access implantation for transpulmonary thermodilution monitoring
8. Severe or terminal renal insufficiency (creatinine clearance <30 ml/min)
9. Severe hepatic insufficiency (hepatic SOFA score>2)
10. Severe cardiac insufficiency, with left ventricular ejection fraction<30%
11. Any history of severe allergic drug reaction (anaphylactic shock or allergic angioedema)
12. Persons deprived of their liberty by a judicial or administrative decision (guardianship or tutelage measure)

6.3 Recruitment procedure

Recruitment will be made at admission of the patients in the 5 listed intensive care units.

	Number of subjects
Total number of subjects to be included	50
Number of sites	5
Enrolment period (months)	7
Number of subjects/site	10
**Number of subjects/site/month**	**1.4**

The recruitment will be performed gradually, stopping temporarily inclusions after each step for DSMB analysis of safety data. Each analysis will be based on a CRF extraction of mortality, mortality during the first five days, serious adverse events, clinical worsening (10.3.2.2.1, p.32) and biological tests. The following schedule will be followed for gradual interruption of inclusions:

- 10 patients (5 in each group)
- 30 patients (15 in each group)

6.4 Termination rules

6.4.1 Criteria and procedures for prematurely terminating the study treatment

As the disease may affect seriously all the organs, and regarding the short duration of the treatment, only anaphylactic reactions during administration of the drug will indicate terminating the treatment prematurely. All other adverse events will be closely monitored by the DSMB, to detect any significant imbalance between groups.

Several situations are possible

- Temporary suspension of treatment: the investigator must document the reason for suspending and resuming the treatment in the participant's source file and the case report form (CRF)

- Premature discontinuation of treatment, but the participant remains enrolled in the study until the end of their participation
- Premature discontinuation of treatment and withdrawal from the study

The investigator must:

- o Document the reason(s)
- o Collect the assessment criteria at the time of ending participation in the study, if the participant agrees
- o Schedule a follow-up for the participant, particularly in case of a serious adverse event.

In the case of severe adverse events, the investigator must notify the sponsor and follow up the participant for 3 months following the premature discontinuation of treatment. Notification of a serious adverse event must be sent by email (eig-vigilance.drc@aphp.fr) to the sponsor. The serious adverse event will be monitored until it is resolved.

6.4.2 Criteria and procedure for premature withdrawal of a participant from the study

- Subjects may exit the study at any time and for any reason.
- The investigator can temporarily or permanently withdraw a subject from the study for any safety reason or if it is in the subject's best interests.
- Subject lost to follow-up: the subject cannot be located. The investigator must make every effort to reconnect with the subject (and record his attempts in the source file), at least to determine whether the subject is alive or dead

If a subject exits the trial prematurely or withdraws consent, any data collected prior to the date of premature exit may still be used.

The case report form must list the various reasons why the subject exited or was withdrawn from the study:

- ☐ Lack of efficacy
- ☐ Adverse reaction
- ☐ Other medical problem
- ☐ Subject's personal reasons
- ☐ Explicit withdrawal of consent
- ☐ Lost to follow-up
- ☐ Death

- ***Treatment stopping rules***:

Each investigator is strongly encouraged to stop administration of the product before the end of the treatment if any of the following events occurs, in the **absence of a clear underlying cause other than treatment administration**:

- Serious bleeding event (necessitating the transfusion of more than 4 packed red cells) in the absence of any invasive procedure.
- Severe diffuse intravascular coagulation, with QT a ratio <20%
- Severe liver cytolysis, with AST and ALT above 10 times normal
- Severe leucopenia (<500 G/L)
- Severe allergic reaction (anaphylactic shock, angio-edema, bronchospasm, severe skin reaction) at the time of or following drug injection
- Severe cardiac arrhythmia at the time of drug injection

6.4.3 Follow-up of participants following premature withdrawal from the study

If a subject exits the trial this will in no way affect the standard care received for his/her condition.
In case of severe adverse events, the investigator must notify the sponsor and monitor the subject for 3 months following the premature termination of treatment. If treatment is stopped prematurely

due to a serious adverse event, a serious adverse event report will be sent by mail (eig-vigilance.drc@aphp.fr) to the sponsor. The serious adverse reaction will be monitored until it is resolved.

6.4.4 Procedures for replacing participants

Subjects exiting the trial will not be replaced. They will receive further standard usual care, and their data will be analysed with intent to treat analysis according to their group of randomization.

6.4.5 Full or partial discontinuation of the study

AH-HP (the sponsor) or the Competent Authority (ANSM) may prematurely discontinue all or part of the trial, temporarily or permanently, upon the recommendation of the Data Monitoring Committee in the following situations:

- first, if suspected unexpected serious adverse reactions (SUSARs) are observed in one of the treatment arms or if there is a discrepancy in the serious adverse reactions between the two treatment arms, requiring a reassessment of the benefit-risk ratio for the trial.
- similarly, AH-HP, as the sponsor, or the Competent Authority (ANSM) may prematurely cancel the trial due to the discovery of unexpected facts or new information about the product, in light of which the objectives of the study or clinical programme are unlikely to be achieved.
- AP-HP, as the sponsor, reserves the right to permanently suspend enrolment at any time if the enrolment targets have not been met.

If the study is cancelled prematurely, AP-HP will inform the Competent Authority (ANSM) and the Institutional Review Board of its decision within 15 days, together with justification for the decision and any recommendations from the Data Monitoring Committee (if applicable). Subjects will receive further standard usual care and will be followed until the end of the study, i.e. 3 months.

7 TREATMENT ADMINISTERED TO STUDY PARTICIPANTS

7.1 Description of the investigational medicinal product(s)

7.1.1 Investigational medicinal product 1

Investigational medicinal product n°1: FX06

FX06, a drug arising from research on fibrin-derived peptide Bβ15-42, is able to stabilize VE-cadherin dependent cell-cell interactions, thereby reducing vascular leak [9,10]. The treatment will be administrated as soon as possible after randomization, but no longer than 12 hours.

FX06 80 mg/ml solution for injection and its placebo will be supplied as clear, colorless single-use glass vials containing 2.75 ml ready-to-use solution, closed with a rubber stopper and aluminum flip-off cap. Each Therapeutic Unit will contain 10 vials.
Treatments will be stored in each ICU in a secured refrigerator, under the supervision of the pharmacy department of each center. The trial medication should be stored at 2–8°C in a secured refrigerator. After storage at 4°C, FX06 80 mg/ml was stable for 24 months.

Composition of FX06 ready to use vials

Ingredient		Function
Peptide $B\beta_{15-42}$		Active ingredient
Disodium hydrogen phosphate, anhydrous	0.118 mg/ml	Buffer
Sodium dihydrogen phosphate, monohydrate	1.265 mg/ml	Buffer

Ingredient		Function
Sodium chloride	4.48 mg/ml	
Water for injection	ad 2.75 ml *	Diluent

* to ensure 2.5 ml retractable volume

The following dosing schedule will be used:
I.V. 200 mg starting dose as a bolus followed by a second bolus of 200 mg 10-15 min later i.v. during 5 days.

	Patient 1	Patient 2	Patient 3	etc
Day 1	2.5 ml	2.5 ml	2.5 ml	
	Wait 10-15 min	Wait 10-15 min	Wait 10-15 min	
	2,5 ml	2,5 ml	2,5 ml	
Day 2	2.5 ml	2.5 ml	2.5 ml	
	Wait 10-15 min	Wait 10-15 min	Wait 10-15 min	
	2,5 ml	2,5 ml	2,5 ml	
Day 3	2.5 ml	2.5 ml	2.5 ml	
	Wait 10-15 min	Wait 10-15 min	Wait 10-15 min	
	2,5 ml	2,5 ml	2,5 ml	
Day 4	2.5 ml	2.5 ml	2.5 ml	
	Wait 10-15 min	Wait 10-15 min	Wait 10-15 min	
	2,5 ml	2,5 ml	2,5 ml	
Day 5	2.5 ml	2.5 ml	2.5 ml	
	Wait 10-15 min	Wait 10-15 min	Wait 10-15 min	
	2,5 ml	2,5 ml	2,5 ml	

400 mg i.v. was the dose used in the FIRE trial [5], and the regiment chosen for the study close to the one used as rescue therapy during EBOLA-induced ARDS [6]. In addition, 6 Covid-19 patients were treated with FX06 as rescue therapy in Frankfurt and Würzburg, Germany up to 7 days (400mg/ day). Four over six patients could improve their condition, while 2 died. No clear adverse event related to the treatment could be detected in this cohort (data submitted for publication).

FX06 and its placebo will be supplied by MChE Handelsges.m.b.H (F4-Pharma) and delivered in each ICU under supervision of the local Pharmacy department of each hospital.

7.1.2 Investigational medicinal product 2

Placebo will be provided also as ready to use vials, containing the vehicle of FX06 drug product, i.e., phosphate-buffered saline. The same packaging as for FX-06 drug will be used for the placebo. The composition is given in the table below

Composition of placebo

Ingredient		Function
Disodium hydrogen phosphate, anhydrous	0.118 mg/ml	Buffer
Sodium dihydrogen phosphate, monohydrate	1.265 mg/ml	Buffer
Sodium chloride	4.48 mg/ml	
Water for injection	ad 2.75 ml *	Diluent

* to ensure 2.5 ml retractable volume

Treatments will be stored in each ICU in a secured refrigerator, as for the treatment.

7.2 Description of Additional medicinal product(s) (treatments required to conduct the study)

None

7.3 Description of traceability elements accompanying the investigational medicinal product(s)

Inclusion and exclusion criteria will be verified by each investigator before inclusion, using a web based questionnaire.

Study treatment will be supply by MChE Handelsges.m.b.H (F4-Pharma). As the Therapeutic units contain 10 vials, In order to track the treatment given to each patient, all the information related to the treatment will be collected on a traceability sheet (Preparations, date and time of administration, Therapeutic Unit number…).

7.4 Authorised and prohibited treatments (medicinal, additional medicinal, surgical), including rescue medications

Adjunctive therapy against COVID-19: As researches are evolving quickly, a specific treatment against SARS-CoV-2 virus will be authorized, as soon as it is clearly stated in a written policy for the hospital center. Particularly, dexamethasone (oral or intravenous) 6 mg daily for up to 10 days or until discharge if sooner will be strongly encouraged, regarding the data from RECOVERY trial (https://www.medrxiv.org/content/10.1101/2020.06.22.20137273v1). Immunosuppressive drugs or monoclonal antibody therapy targeting the inflammation process will not be authorised.

Standard usual care will be provided in both groups:

- **Management of ARDS** will follow last recommendations from the French society of intensive care (SRLF-Société de reanimation de langue française): https://www.srlf.org/rfe-srlf-prise-en-charge-du-syndrome-de-detresse-respiratoire-aigue-sdra-de-ladulte-a-la-phase-initiale/
    - Tidal volume will be set at 6 ml/kg of ideal body weight
    - Pplat will be monitored continuously, and kept ≤ 28 cmH_2O
    - PEEP (positive end-expiratory pressure) will be the highest to reach a plateau pressure of 28 cmH_2O
    - Neuromuscular blockers will be added during 48h in case of PaO_2/ FiO_2 ratio below 150 mmHg, and reevaluated afterwards
    - Prone positioning (≥16 hrs) will be encouraged if the PaO_2/ FiO_2 ratio is below 150 mmHg
    - Contact with an ECMO center will be encouraged in case of a PaO_2/ FiO_2 ratio below 80 mmHg or impossibility to maintain a protective mechanical ventilation, despite those measures
    - CO2 removal devices will be discouraged
    - Nitric oxide administration will be discouraged
- Administration of fluids will be protocolized during the first 72h

Crystalloids will be allowed in case of circulatory failure with signs of pre-load dependence. The solution used will be NaCl 0.9%, and ringer lactate encouraged after 2 litters.

- Packed red cells will be allowed if Hb<7g/dl or active bleeding. Fresh frozen plasma if thromboplastin time ratio<20% or if severe bleeding.
- Platelets will be allowed if <30 G/L or in case of severe bleeding.
- Albumin will not be allowed, as its efficacy is not demonstrated in this pathology, and neither in a large randomized trial in SIRS associated with sepsis [17].
- Adjunctive hydrocortisone and fludrocortisone will be discouraged, as its efficacy has not been demonstrated to date in this pathology.

7.5 *Methods for monitoring compliance with the treatment*

Treatment actually administered to the patient will be assessed by the investigator at the end of the perfusion and recorded in the e-CRF.

8 EFFICACY ASSESSMENT

8.1 *Description of efficacy endpoints assessment parameters*

Extravascular lung water index (EVLWi) measured by transpulmonary thermodilution. See par 4.1

8.2 *Anticipated methods and timetable for measuring, collecting and analysing the efficacy data*

EvLWi recorded between D1 and D7. See par 5.5

9 SPECIFIC STUDY COMMITTEES

NB: the Data Safety Monitoring Board (DSMB) is described in Safety section.

9.1 *Steering Committee*

- Members of the committee:
  - Dr Bréchot (Coordinating investigator)
  - Project manager URC et DRCI (sponsor's appointed representatives for the trial)
  - Dr Hajage (methodologist)
- Missions:
  - Define the overall structure of the study, coordinate information, determine the initial methodology and oversee the trial. Propose procedures to be followed during the study. The sponsor (APHP) retains the decision-making authority.

10 SAFETY ASSESSMENT - RISKS AND BURDEN ADDED BY THE STUDY

10.1 *Description of Safety endpoints assessment parameters*

Safety endpoints will overlap with efficacy (see par 4.1). Coagulation disorders will be specifically monitored, as coagulation might theorically be affected by the drug, although this effect was not found in safety studies (see investigator brochure).

10.2 *Anticipated methods and timetable for measuring, collecting and analysing the safety endpoints*

See par 4.1

10.3 *Recording and reporting adverse events*

10.3.1 Definitions

According to Article R1123-46 of the French Public Health Code:

- **Adverse event**

Any untoward medical occurrence in a trial subject, which does not necessarily have a causal relationship with the clinical trial or with the investigational product.

- **Adverse reaction to an investigational medicinal product**

Any adverse event occurred in a trial subject, which has a causal relationship with the clinical trial or with the investigational medicinal product

- **Serious adverse event or reaction**

Any adverse event or reaction that at any dose of medication, results in death, threatens the life of the research subject, requires hospitalisation or prolongs hospitalisation, causes a severe or long-term disability or handicap, or results in a congenital abnormality or deformity.

- **Unexpected adverse reaction to an investigational medicinal product**

Any adverse reaction to the product, whose nature, severity, frequency or outcome is inconsistent with the safety information described in the Reference Safety Information (summary of product characteristics, or the investigator's brochure if the product is not authorised).

According to Article R.1123-46 of the Code de la Santé Publique and the guidelines for clinical trial sponsors (ANSM):

- **Emerging safety issue**

Any new safety information that may lead to a reassessment of the risk/benefit ratio of the trial or the investigational medicinal product, modifications in the investigational medicinal product use, the conduct of the clinical trial, or the clinical trial documents, or a suspension, interruption or modification of the protocol of the clinical trial or other similar trials.
For the clinical trials involving the first administration or use of an investigational medicinal product in healthy volunteers, any serious adverse reaction.
Examples:
a) any clinically significant increase in the frequency of an expected serious adverse reaction
b) suspected unexpected serious adverse reactions in patients who have terminated their participation in the clinical trial that are notified by the investigator to the sponsor together with follow-up reports
c) any new safety issue relating to the conduct of the clinical trial or the development of the investigational medicinal product, that may impact the safety of the trial subjects.
Examples:

- a serious adverse event likely to be related to the interventions and the trial's diagnostic procedures and which may impact the conduct of the clinical trial,
- a significant risk on the trial subjects such as ineffectiveness of the investigational medicinal product in treating a life-threatening illness under investigation,
- significant safety results from a recently completed non-clinical study (such as a carcinogenicity study),
- the premature termination, or temporary suspension, of a trial conducted on the same investigational medicinal product in another country, for safety reasons,
- an unexpected serious adverse reaction associated with a non-experimental medication required for the conduct of the clinical trial, (e.g. challenge agents, rescue treatment)

d) recommendations from the Data Safety Monitoring Board (DSMB), if applicable, that may affect the safety of the trial subjects
e) any suspected unexpected serious adverse reaction (SUSAR) reported to the sponsor by another sponsor of a trial carried out in a different country but relating to the same medication.

10.3.2 The role of the investigator

The investigator must **assess the seriousness criteria of each adverse event** and record all serious and non-serious adverse events in the case report form (eCRF)
The investigator must **document** serious adverse events **as thoroughly as possible** and provide a definitive medical diagnosis, if possible.

The investigator must **assess the severity** of the adverse events by using a CTCAE scale:

- ○ -

- or a severity grading scale for adverse events, attached to the protocol

The investigator must assess the **causal relationship** between the serious adverse events and the investigational medicinal product.

The method used by the investigator is based on the WHO Uppsala Monitoring Centre method and uses the following causality terms:

- Certain
- Probable/likely
- Possible
- Unlikely (not ruled out).

These terms are defined as follows (extracted from the WHO-UMC causality categories, version dated 17/04/2012).

Table: WHO-UMC causality categories (extract)

**Causality term**	Assessment criteria*
**Certain**	· Event or laboratory test abnormality, with plausible time relationship to drug intake **<br>· Cannot be explained by disease or other drugs<br>· Response to withdrawal plausible (pharmacologically, pathologically)<br>· Event definitive pharmacologically or phenomenologically (i.e. an objective and specific medical disorder or a recognized pharmacological phenomenon)<br>· Rechallenge satisfactory, if necessary
**Probable / Likely**	· Event or laboratory test abnormality, with reasonable time relationship to drug intake**<br>· Unlikely to be attributed to disease or other drugs<br>· Response to withdrawal clinically reasonable<br>· Rechallenge not required
**Possible**	· Event or laboratory test abnormality, with reasonable time relationship to drug intake **<br>· Could also be explained by disease or other drugs<br>· Information on drug withdrawal may be lacking or unclear
**Unlikely**	· Event or laboratory test abnormality, with a time to drug intake **<br>· that makes a relationship improbable (but not impossible)<br>· Disease or other drugs provide plausible explanations

*All points should be reasonably complied with
** Or study procedures

10.3.2.1 Serious adverse events that require the investigator to notify the sponsor without delay

As per article R.1123-49 of the *Code de la Santé Publique* (French Public Health Code), the investigator notifies the sponsor without delay (and at the latest within 24 hours) on the day the investigator becomes aware of any serious adverse event which occurs during a study that meets the description in line 1° of Article L.1121-1 of the Code de la Santé Publique, with the exception of any event which is listed in the protocol and, if applicable, in the investigator's brochure as not requiring notification.

A serious adverse event is any untoward medical occurrence that:

1- results in death<br>2- is life-threatening to the participant enrolled in the study<br>3- requires hospitalisation or prolongation of existing hospitalisation<br>4- results in persistent or significant disability/incapacity

5- is a congenital anomaly/birth defect

It should be noted that the sponsor will particularly monitor the frequency of deaths occurring during the first 5 days (it concerns less than 25 % of the deaths in a prospective cohort of 36 patients meeting criteria for inclusion (18).

10.3.2.2 Specific features of the protocol

10.3.2.2.1 Other events that require the investigator to notify without delay the sponsor

- Adverse events judged as being "medically significant" not listed in the normal and natural course of the condition (see below)
  - Major bleeding (bleeding necessitating a transfusion of more than 4 packed red blood cells), in the absence of invasive procedure
  - Anaphylactic shock at the time of drug injection
  - Clinical worsening: rescue with VV-ECMO

The investigator must notify the sponsor **without delay (and at the latest within 24 hours) on the day when the investigator becomes aware** of these adverse events, according to the same modalities and within the same timeline as for serious adverse events (see above).

- **In utero exposure**

The investigator must notify the sponsor without delay on the day the investigator becomes aware of any pregnancy that occurs during the study, even if it is not associated with an adverse event.
If the investigational medicinal product is genotoxic, every case of maternal or paternal exposure must be notified.
Notify using a specific form appended to the protocol.

- Exposure **while breastfeeding**

Exposure while breastfeeding occurs if an infant or a child may have been exposed to a medicinal product via the breast milk of a mother being treated with an investigational medicinal product.
Even if it is not associated with an adverse event, the investigator must notify the sponsor without delay on the day the investigator becomes aware of the exposure while breastfeeding.

10.3.2.2.2 Serious adverse events that do not require the investigator to notify the sponsor without delay

These serious adverse events are simply recorded in the case report form. A CRF extraction of these serious adverse events will be realized according to Data Safety Monitoring Board (DSMB) or AP-HP's Safety department's request.

- ***Normal and natural course of the condition*:**

SARS-CoV-2 induced ARDS is an extremely severe condition, with up to 60% ICU mortality [3]. Moreover, the condition, consisting in severe hypoxia, may affect all organs.
Serious adverse events linked with the condition include particularly:

- Hemodynamic worsening
- Liver cytolysis/insufficiency
- Acute renal failure
- Diffuse intra-vascular coagulation/bleedings
- Leucopenia/thrombopenia/anemia
- Severe mesenteric ischemia
- Brain death/hemorragic stroke/ischemic stroke
- Pulmonary embolism

- Acute myocardial infarction/ cardiac rhythm disorders
- Aspiration pneumonia/ ventilator acquired pneumonia/ bacteraemia/fungemia

In this context, those disorders (except deaths) do not need to be notified to the sponsor without delay. A CRF extraction of those serious adverse events will be realized according to a frequency defined in the DSMB charter. A particular monitoring of coagulation tests will be also conducted, to detect any imbalance between groups. These data will be sent to the Data Safety Monitoring Board members and the safety department at expertisecsi.drc@aphp.fr. If there is any imbalance between the randomization groups affecting the safety of trial subjects and which requires the sponsor to take urgent safety measures, the ANSM will be informed about the emerging safety issue without delay.

- ***Adverse events during the trial possibly related with the treatments prescribed as part of the patient's standard care***

The investigator must report these events to his *Centre Régional de Pharmacovigilance* (CRPV).

10.3.2.3 Period during which SAEs must be notified without delay by the investigator to the sponsor

The investigator notifies the sponsor without delay (and at the latest within 24 hours) of all the serious adverse events listed in the corresponding section:

- starting from the date on which the subject begins treatment with the investigational medicinal product
- throughout the whole follow-up period required for the trial
- until 4 weeks after the end of the subject's treatment with the investigational medicinal product.
- after the end of the clinical trial, if the SAE is likely to be due to the investigational medicinal product (IMP) (e.g. serious reactions that could appear at long term after exposure to the medication, such as cancers or congenital abnormalities) . In that case, the investigator does not have to systematically and indefinitely collect all SAEs possibly related to the IMP, but must notify all possible SAEs related to the IMP of which he has knowledge.

10.3.2.4 Procedures and deadlines for notifying the sponsor

The investigator should initially complete a SAE reporting form (contained in the case report form). The investigator completes the SAE report form in the e-CRF, then validates, prints and signs the form before sending it by email; The form is then included in the patient’s medical chart. In case of failure to connect to the e-CRF, the investigator should complete, sign and send the SAE report form to the safety Department. As soon as the connection is restored, the investigator must complete the SAE report form in the e-CRF.

Initial form must comprise:

- precise description of the case,
- SAE classification as expected or not
- SAE intensity
- SAE precise date of occurrence and resolution
- measures undertaken and treatments added
- whether the treatment was interrupted or not
- its evolution
- Causal relationship between the SAE and the investigational drug.
- Causal relationship between the SAE and the underlying condition/other treatments administrated.

Whenever possible, the investigator will provide the sponsor with any documents that may be useful for medical assessment of the case (medical reports, laboratory test results, results of additional exams, etc.). These documents must be anonymized. In addition, the investigator must state the study acronym and the number and initials of the study participant on each paper.

Any adverse event will be monitored until fully resolved (stabilisation at a level considered acceptable by the investigator or return to the previous state) even if the subject has terminated his participation in the trial.

The initial report, the SAE follow-up reports and all other documents must be sent to the sponsor's safety Department by mail (eig-vigilance.drc@aphp.fr). It is possible to send the SAE to the AP-HP's Safety department by fax No. **+33 (0)1 44 84 17 99** only in case of unsuccessful attempt to send the SAE by e-mail. This is to avoid duplicated reports.

For trials which use e-CRF

- the investigator completes the SAE report form in the e-CRF, then validates, prints and signs the form before sending it by mail;
- In case of failure to connect to the e-CRF, the investigator should complete, sign and send the SAE report form to the safety Department. As soon as the connection is restored, the investigator must complete the SAE report form in the e-CRF.

The investigator must comply with all requests for additional information from the sponsor.
For all questions relating to an adverse event report, the safety Department can be contacted via email at vigilance.drc@aphp.fr.

For cases of in utero exposure, the investigator will complete the "Notification and Follow-up form for a pregnancy occurring during participation in a study".
The investigator must monitor the pregnant woman throughout her pregnancy or until the pregnancy is terminated, and must notify the sponsor of the outcome of the pregnancy using this form.
If the outcome of the pregnancy falls within the definition of a serious adverse event (miscarriage, pregnancy termination, foetal death, congenital abnormality, etc.), the investigator must follow the procedure for reporting SAEs.
The initial pregnancy notification, the SAE follow-up reports, and any other documents will be sent to the sponsor according to the same procedures specified herein.
If it was the father who was exposed, the investigator must obtain the pregnant woman's permission before collecting information about the pregnancy.

10.3.3 Role of the sponsor

The sponsor, represented by its Safety Department, shall continuously, throughout the trial, assess the safety of each investigational medicinal product throughout the study.

10.3.3.1 Analysis and declaration of serious adverse events

The sponsor assesses:

- the **seriousness** of all reported adverse events,
- the **causal relationship** between these adverse events and investigational medicinal product and any other treatments,
  All serious adverse events for which the investigator and/or the sponsor suspect a causal relationship with the investigational medicinal product are classed as suspected serious adverse reactions.
- the **expectedness assessment** of the serious adverse reactions
  Any serious adverse reaction whose nature, severity, frequency or outcome is inconsistent with the safety information described in the investigator's brochure, is considered unexpected.
  The sponsor, acting through its safety Department, assesses the expectedness of the serious adverse reaction based on the information described below**.**

❖ For serious adverse events likely to be related to the investigational medicinal product(s):
- refer to the Investigator's Brochure

- For the serious adverse events likely to be related to blood samples : malaise, vomiting, pain, hematoma

The sponsor will report all suspected unexpected serious adverse reactions (SUSARs), within the regulatory time frame, to the ANSM (French Health Products Safety Agency)).
- The sponsor must send the initial report without delay upon receipt of the unexpected serious adverse reaction if it is fatal or life-threatening, or otherwise within 15 days from receipt of any other type of unexpected serious adverse reaction;
- The sponsor must provide all relevant additional information by sending follow-up reports, within 8 calendar days following receipt.

Any suspected unexpected serious adverse reaction must also be declared electronically using the Eudravigilance European adverse drug reactions database managed by the European Medicines Agency (EMA).

The sponsor must notify all the investigators about any information that could adversely affect the safety of the trial subjects.

Special rules for double blind trials
After unblinding, if the administered product is the investigational medicinal product, the case will be immediately reported as suspected serious unexpected adverse reaction (SUSAR); however, if the administered product is the comparator, the unexpected nature of the adverse reaction will be re-assessed according to the reference document of the comparator found in the protocol.

10.3.3.2 Analysis and declaration of other safety data

This relates to any new safety data that may lead to a reassessment of the risk/benefit ratio of the trial or the investigational medicinal product, modifications in the investigational medicinal product use, the conduct of the clinical trial, or the clinical trial documents, or a suspension, interruption or modification of the protocol of the clinical trial or other similar trials.

The sponsor will inform the competent authority and the Ethics committee without delay after becoming aware of the emerging safety issue and, if applicable, describe which measures have been taken.

Following the initial declaration of emerging safety issue, the sponsor will declare to ANSM any additional relevant information about the new safety issues in the form of a follow-up report, which must be sent no later than 8 days after becoming aware of the information.

If the suspected unexpected serious adverse reaction meets the definition of an emerging safety issue, the sponsor will report both the SUSAR and the emerging safety issue to the ANSM according to the appropriate modalities and within the regulatory timelines as previously described.

10.3.3.3 Annual safety report

Not applicable taking into account the total duration of the research (5 months) less than 1 year.

10.3.4 Data Safety Monitoring Board (DSMB)

A DSMB will be set up for this trial by the sponsor. The DSMB must hold its first meeting before the first subject is enrolled and ideally before the protocol is submitted to the competent authority and the CPP (Research Ethics Committee). The DSMB’s preliminary meeting will take place before enrolment, to define the frequency of committee meetings and safety data required.

All missions as well as the specific operating procedures of the DSMB are described in the study's DSMB charter.
The members of the DSMB are:

- 2 intensivists: Pr Jean CHASTRE (APHP, Sorbonne Université, Paris), Pr Peter RADERMACHER, Ulm university medical school, Germany)
- 1 méthodologist : Florence CANOUI-POITRINE

The DSMB will operate in accordance with the sponsor's procedures. The DSMB works in an advisory capacity only and the sponsor retains all decision-making authority.

The recruitment will be performed gradually, stopping temporarily inclusions after each step for DSMB analysis of safety data. Each analysis will be based on a CRF extraction of mortality, mortality during the first five days, serious adverse events, clinical worsening and biological tests (NFS, TP, TCA, Fibrinogen, creatininemia, liver tests (AST, ALT) during first 7 days.

11 DATA MANAGEMENT

11.1 *Data collection procedures*

During and after the clinical study, all data will be collected by the investigator and others specialised collaborators.

11.2 *Identification of data recorded directly in the CRFs which will be considered as source data*

Chest X-Rays results

11.3 *Right to access data and source documents*

11.3.1 Data access

In accordance with GCPs:
- the sponsor is responsible for ensuring all parties involved in the study agree to guarantee direct access to all locations where the study will be carried out, the source data, the source documents and the reports, for the purposes of the sponsor's quality control and audit procedures or inspections by the competent authority
- the investigators will ensure the persons in charge of monitoring, quality control and auditing or inspecting the interventional study involving human participants have access to the documents and personal data strictly necessary for these tasks, in accordance with the statutory and regulatory provisions in force (Articles L.1121-3 and R.5121-13 of the French Public Health Code)

11.3.2 Source documents

The source documents are any original document or item that proves the existence or accuracy of a data-point or fact recorded during the trial. Source documents will be kept by the investigator, or by the hospital in the case of hospital medical records, for the statutory period.

Document sources will be electronic medical records from both ICUs, laboratory tests results and imaging reports from La Pitié-Salpêtrière hospital facilities.

11.3.3 Data confidentiality

The persons responsible for the quality control of clinical studies (Article L.1121-3 of the French Public Health Code) will take all necessary precautions to ensure the confidentiality of information relating to the investigational medicinal products, the study, the study participants and in particular the identity of the participants and the results obtained.
These persons, as well as the investigators themselves, are bound by professional secrecy (in accordance with the conditions set out in Articles 226-13 and 226-14 of the French Criminal Code).
During and after the clinical study, all data collected about the study participants and sent to the sponsor by the investigators (or any other specialised collaborators) will be anonymized.

Under no circumstances shall the names and addresses of the participants involved be shown.
The subject's initials will be recorded, along with an identification code specific to the study indicating the order of enrolment.

The sponsor will ensure that each subject has given written permission for any personal information about him or her which is strictly necessary for the quality control of the study to be accessed.

11.4 Data processing and storage of research documents and data

11.4.1 Identification of the data processing manager and location(s)

Data management and analysis of the data will be performed under the responsibility of the Clinical Research Unit of La Pitié-Salpêtrière hospital/Charles Foix by Dr HAJAGE David. The data will be collected in the eCRFs by clinical study technicians and investigators.

11.4.2 Data entry

Non-identifying data will be entered electronically via a web browser, by the investigator in charge of the patient or a research technician.

11.5 Data ownership

AP-HP is the owner of the data. The data cannot be used or disclosed to a third party without its prior permission.

12 STATISTICAL ASPECTS

12.1 Description of statistical methods to be used including the timetable for the planned interim analyses

The statistical analysis will be performed by a biostatistician of the Clinical Research Unit of Pitié-Salpêtrière Hospital, under the responsibility of Dr David HAJAGE.

Statistical analyses will be performed with the SAS® or R software with the most recent version at the time of the analysis.

Descriptive analysis will always be overall and by treatment group.

Unless specially mentioned, comparative analysis between groups will be systematically performed without adjustment (univariate analysis);

Flow chart
A flow chart according to the CONSORT recommendations will describe the recruitment and the follow-up of all study population.

Study population
The primary analysis of efficacy endpoints will be performed on all randomized patients for whom a baseline evaluation is available, on an intention-to-treat (ITT) population. All patients will therefore be analyzed in their initial randomization groups.
The safety set is defined as all randomized patients having received at least one dose of study treatment. Safety endpoints will be analyzed on the safety set.

Descriptive analysis
Qualitative variables will be described with frequency and percentage distributions, and quantitative variables with median, interquartile range, minimum and maximum values, mean and standard deviation.
Censored data will be described by Kaplan-Meier estimation or cumulative incidence function, with rate at D7 and D30 with 95% confidence interval.

Baseline characteristics
Demographical, clinical, biological and treatment characteristics will be described.

Primary analysis
The primary analysis will be performed in the intention to treat population.

The primary outcome is the change in EVLWi between D1 and day 7. These changes will be compared between both arms using analysis of covariance (ANCOVA), adjusted for the D1 EVLWi. This is fully equivalent to comparing EVLWi scores at D7 adjusted for the baseline EVLWi. Results will be expressed in terms of adjusted mean changes, with their 95% confidence interval. The unadjusted supportive analysis will be carried out by comparing EVLWi at day 7 between the two arms with a t test.
If the distribution of EVLWi is clearly incompatible with the ANCOVA or t test assumptions, then these analyses will be conducted on the ranks.
For patients dying before 7 days, the last thermodilution data will be retained for the primary analyses. If the patient's condition no longer warrants the presence of the transpulmonary thermodilution systems, then the catheter is removed before 7 days. In this case, the measurement of EVLWi can no longer be made, the last thermodilution data will be retained for the primary analyses. A sensitivity excluding those patients will be performed for analysing the primary endpoint.

Secondary analyses

For safety, the types and severity of AEs and SAEs will be described as frequency and percent by treatment arm. Fisher's exact tests will be used to compare the two treatment arms in terms of proportion of patients with death (all cause), at least one AE/SAE, at least one SAE. The number of these events (except death) will be compared between arms by Poisson regression.

Overall survival at D30 will be compared between the two groups using a logrank test.

The evolution of EVLWi during 7 days and other longitudinal repeated efficacy secondary outcomes will be compared between the two groups using a linear mixed effect model to take into account within subject correlation. If the model assumptions (normally distributed residuals with constant variance) is debatable, these analyses will be conducted on the ranks.

For other efficacy secondary outcomes, data will be described graphically and compared between arms by:

- chi2 test or fisher test for binary outcomes;
  t-test or wilcoxon rank-sum test for quantitative variables.

Subgroups analyses will be performed according to :

  - Presence of VV-ECMO at baseline (Yes/No)
  - EVLWi at baseline (>10 ml/kg) (Yes/ No)

12.2 Calculation hypotheses for the number of participants required and the result

According to previous studies, standard deviation for EVLWi during ARDS is around 5 ml/kg predicted body weight [2]. Assuming a baseline mean of EVLWi of 13.4 and a 30% decrease in EVLWi in FX06 treated patients compared to controls at Day 7, **for 80% power and an overall 5% two-sided α-risk,** the required sample size is **25 patients per group.**

12.3 Anticipated level of statistical significance

Analyses will be performed with a two-sided alpha risk of 5%.

12.4 Statistical criteria for termination of the study.

No interim analysis will be performed.

12.5 Method for taking into account missing, unused or invalid data

Missing data will be described.
No replacement of missing patients is planned. Dealing with very severe patients hospitalized in ICU, we anticipate no loss of follow up.

12.6 Management of modifications made to the analysis plan for the initial strategy.

Any changes made to the statistical analysis plan must be justified and will be done before the beginning of the statistical analysis.

13 QUALITY CONTROL AND ASSURANCE

Every research project involving human participants managed by AP-HP is ranked according to the projected risk incurred by study participants using the classification system specific to AP-HP sponsored interventional research involving human participants.

13.1 General organisation

The sponsor must ensure the safety and respect of individuals who have agreed to participate in the study. The sponsor must implement a quality assurance system to best monitor the implementation of the study in the investigation centres.

For this purpose, the sponsor shall assign Clinical Research Associates (CRA) whose primary role is to carry out regular follow-up visits at the study locations, after having carried out the initial visits.
The purpose of monitoring the study, as defined in Good Clinical Practices (GCP section 5.18.1), is to verify that:

- the rights, safety and protection of the research participants are met
- the data reported are exact, complete and consistent with the source documents
- the study is carried out in accordance with the protocol in force, the GCPs and the statutory and regulatory provisions in force

13.1.1 Strategy for centre opening

The strategy for opening the centres established for this study is determined using the appropriate monitoring plan.

13.1.2 Scope of centre monitoring

In the case of this D risk study, the appropriate monitoring level has been determined based on the complexity, the impact and the budget for the study. Therefore, in agreement with the coordinating investigator, the sponsor has determined the logistical score and impact resulting in a study monitoring level to be implemented: level: **high level**.

13.2 Quality control

A Clinical Research Associate (CRA) appointed by the sponsor will be responsible for the good completion of the study, for collecting, documenting, recording and reporting all handwritten data, in accordance with the Standard Operating Procedures applied within the DRCI (Clinical Research and Innovation Department) and in accordance with Good Clinical Practices as well as the statutory and regulatory requirements.

The investigator and the members of the investigator’s team agree to make themselves available during regular Quality Control visits carried out by the Clinical Research Associate. During these visits, the following elements will be reviewed depending on the monitoring level:

- written consent
- compliance with the study protocol and with the procedures defined therein
- quality of the data collected in the case report form: accuracy, missing data, consistency of the data with the “source” documents (medical files, appointment books, original copies of laboratory results, etc.)
- management of the treatments used

13.3 Case report forms

All information required by the protocol must be entered in the case report forms. The data must be collected as and when it is obtained, and clearly recorded in these case report forms. Any missing data must be coded.
Every site will have access to the electronic case report forms via a web-based data collection system. Investigators will be given instructions for using this tool.
Using on-line case report forms means the CRA can view the data quickly and remotely. The investigator is responsible for the accuracy, quality and relevance of all the data entered. In addition,

there are consistency checks to ensure the data are verified immediately upon being entered. The investigator must validate any changes to the values in the case report form. An audit trail will be kept of all changes. A justification can be added when applicable, as a comment. A print-out, authenticated (signed and dated) by the investigator, will be requested at the end of the study. The investigator must archive a copy of the authenticated document that was delivered to the sponsor.

13.4 Management of non-compliances

Any events that occur as a result of non-compliance – by the investigator or any other individual involved in running the study – with the protocol, standard operating procedures, good clinical practices or statutory and regulatory requirements must be recorded in a declaration of non-compliance and sent to the sponsor.

These non-compliances will be managed in accordance with the sponsor's procedures.

13.5 Audits/inspections

The investigators agree to consent to the quality assurance audits carried out by the sponsor as well as the inspections carried out by the competent authorities. All data, documents and reports may be subject to regulatory audits and inspections. These audits and inspections cannot be refused on the grounds of medical secrecy.

An audit can be carried out at any time by individuals appointed by the APHP and independent of those responsible for the research. The aim of the audits is to ensure the quality of the study, the validity of the results and compliance with the legislation and regulations in force.

The persons who manage and monitor the study agree to comply with the sponsor's requirements and with the competent authority regarding study audits or inspections.

The audit may encompass all stages of the study, from the development of the protocol to the publication of the results, including the storage of the data used or produced as part of the study.

13.6 Principal Investigator's commitment to assume responsibility

Before starting the study, each investigator will give the sponsor's representative a copy of his/her updated personal *curriculum vitæ*, signed and dated less than one year, with his/her RPPS number (*Répertoire Partagé des Professionnels de Santé*, Collective Database of Health Professionals). The CV must include any previous involvement in clinical research and related training.

Each investigator will commit to comply with legislation and to conduct the study in line with GCP, in accordance with the Declaration of Helsinki.

The Principal Investigator at each participating site will sign a commitment of responsibility (standard DRCI document) which will be sent to the sponsor's representative.

The investigators and their staff will sign a delegation of duties form specifying each person's role and will provide their CVs.

14 ETHICAL AND LEGAL CONSIDERATIONS

14.1 Methods for informing research participants and obtaining their consent

In accordance with Article L.1122-1-1 of the French Public Health Code, no research can be carried out on a person without his/her free and informed consent, obtained in writing after the person has been given the information specified in Article L.1122-1 of said Code.

If the person is unable to give his or her written consent, consent may be obtained, in descending order of priority, from a legal representative, family member or a close relative. These persons must have no connection whatsoever to the investigator or the sponsor.

Therefore, the site investigator will obtain consent for inclusion in FX-COVID (as approved by local IRBs) from a legal representative, family member or a close relative prior to randomization. The investigator will retain the original signed and dated consent form. The research subject relative will keep a signed and dated copy of the consent form.

Should a close relative be absent, eligible patients will be randomized according to the specifications of emergency consent (Article L.1122-1-3 CSP) after IRB approval. The patient's legal representative will be informed as soon as possible of the inclusion and asked to give his or her written consent. The patient will be informed and asked to give his/her consent for the continuation of the trial when his/her condition will allow during the subject's study participation. Investigators should to indicate in the medical record the inclusion of a patient unable to consent in emergency situations in the research protocol.

Formulates of consent form will be: legal representative/family member/close relative of patient when patient is unable to sign consent; patient for authorization for pursuit of research, legal representative/family member/close relative of patient for pursuit.

When eligible patients will not be randomized, the reason for non randomization (lack of consent from substitute decision maker; refusal from attending physician; prior or current enrolment in a confounding RCT, patient missed by the research team) will be recorded in the screening log file.

14.2 Prohibition from participating in another clinical study or exclusion period set after the study

Whilst participating in this trial, subjects may not take part in any other interventional clinical study without first speaking to the doctor in charge of this trial. The participants can however participate in other non-interventional studies

14.3 Compensation for participants

No compensation will be paid to patients included in this trial.

14.4 Registration on the National Register of study participants to studies involving human participants concerning the products mentionned in Article L. 5311-1 of the Code de la santé publique

Not applicable

14.5 Authorisation for the research location

The study will be carried out in treatment units on individuals presenting a clinical condition for which the units are specialised and who require interventions that are routinely performed at those units. Therefore, the research location does not require specific authorisation.

14.6 Legal obligations

14.6.1 Role of the sponsor

Assistance Publique - Hôpitaux de Paris (AP-HP) is the sponsor of this study and, by delegation, the DRCI (Clinical Research and Innovation Department) carries out the study's missions in accordance

with Article L.1121-1 of the *Code de la Santé Publique (French Public Health Code)*. Assistance Publique - Hôpitaux de Paris reserves the right to halt the study at any time for medical or administrative reasons. In this case, notification will be sent to the investigator.

14.6.2 Request for approval from the CPP (Research Ethics Committee)

Prior to starting the study, AP-HP, as sponsor, must obtain for this interventional study involving human participants concerning a medicinal product for human use, approval from the appropriate CPP (Research Ethics Committee), within the scope of its authority and in accordance with in force legislation and regulatory requirements.

14.6.3 Request for authorisation from ANSM

Prior to starting the study, AP-HP, as sponsor, must obtains authorisation from the ANSM (French Health Products Safety Agency) for the interventional study involving human participants concerning a medicinal products for human use, within the scope of the ANSM's authority and in accordance with in force legislation and regulatory requirements.

14.6.4 Procedures relating to data protection regulations

The computer file used for this research is implemented in accordance with French (amended "Informatique et Libertés" law governing data protection) and European (General Data Protection Regulation – GDPR) regulations.

- Request for autorisation by the CNIL (French Data Protection Agency)

This research is not governed by the CNIL "Reference Method" (MR-001) because emergency consent and unability to obtain a written consent at inclusion.
The sponsor must obtain the authorisation of the CNIL (French Data Protection Agency) before implementing any data processing involving the data required to conduct the research.

14.6.5 Amendments to the research

Any substantial modification to the protocol by the coordinating investigator must be sent to the sponsor for approval. After approval is given, the sponsor must obtain, approval from the CPP (Research Ethics Committee) and authorisation from the ANSM within the scope of their respective authorities, before the amendment can be implemented.
The information note and the consent form can be revised if necessary, in particular in case of a substantial amendment to the study or if adverse reactions occur.

14.6.6 Final study report

The final report for the research involving human participants referred to in Article R1123-67 of the *Code de la Santé Publique* (French Public Health Code) is written and signed by the sponsor and the investigator. A report summary drafted according to the reference plan of the competent authority must be sent to the competent authority within a period of one year following the end of the study, i.e., the end of the participation of the last participant in the study.

14.6.7 Archiving

Specific documents for an interventional study involving human participants concerning a medicinal product for human use will be archived by the investigator and the sponsor for 15 years after the end of the research.
This indexed archiving includes, in particular:

- A sealed envelope for the investigator containing a copy of all the information notes and consent forms signed by all individuals at the centre who participated in the study;
- A sealed envelope for the sponsor, containing a copy of all the information notes and consent forms signed by all individuals at the centre who participated in the study;

- "Study" binders for the Investigator and the sponsor, including (non-exhaustive list) :
  - the successive versions of the protocol (identified by the version number and its date), and any appendices
  - the ANSM authorisations and CPP (Research Ethics Committee) decisions
  - any correspondence
  - the enrolment list or register
  - the appendices specific to the research
  - final study report
- The data collection documents

15 FUNDING AND INSURANCE

15.1 *Funding sources*

- Bouygues SA, as donation
- Sanofi SA, as donation
- MChE Handelsges m.b.H (F4-Pharma), Vienna, Austria

15.2 *Insurance*

For the duration of the study, the Sponsor will take out an insurance policy covering the sponsor's own public liability, as well as the public liability for all the physicians involved in the study. The sponsor will also provide full compensation for any damages caused by the study to the participant enrolled and their beneficiaries, unless the sponsor can prove that the harm is not the fault of the sponsor or any collaborator. Compensation cannot be refused on the grounds of a third-party act or the voluntary withdrawal of the person who initially consented to participate in the study.

Assistance Publique-Hôpitaux de Paris (AP-HP) has taken out insurance with HDI-GLOBAL SE through the insurance broker BIOMEDIC-INSURE for the full study period, which covers its own public liability and that of any collaborator (physician or research staff), in accordance with Article L.1121-10 of the *Code de la Santé Publique* (French Public Health Code).

16 PUBLICATION RULES

The author(s) of any publication relating to this study must include the AP-HP among their affiliations and name the sponsor AP-HP (DRCI) and the source of funding, if funded by a call for tenders (e.g. national or regional PHRC); a copy of the publication must be sent to the sponsor (see below for rules governing affiliation, and naming the sponsor and funders).

16.1 Mention of AP-HP affiliation for projects sponsored by AP-HP

- If an author has several affiliations, the order in which the institutions are mentioned (AP-HP, University, INSERM, etc.) is not important
- However, if the trial is funded by an internal call for tenders at the AP-HP, the first affiliation must be "AP-HP"
- Each of these affiliations must be identified by an address and separated by a semicolon

The AP-HP institution must feature under the acronym "**AP-HP**" first in the address, specifically followed by: **AP-HP**, hospital, department, city, postcode, France

16.2 Mention of the sponsor AP-HP (DRCI) in the acknowledgements of the text

- “The sponsor was Assistance Publique – Hôpitaux de Paris (Direction de la Recherche Clinique et de l’Innovation)”

16.3 Mention of the financial backer in the acknowledgements of the text

- Others call for tenders: “The study was funded by donation from Bouygues SA, Sanofi SA, MChE Handelsges m.b.H (F4-Pharma), Vienna, Austria

This study has been registered on the website http://clinicaltrials.gov/ under number

17 BIBLIOGRAPHY

1. Matthay MA, Zemans RL. The acute respiratory distress syndrome: pathogenesis and treatment. Annu Rev Pathol. 2011;6: 147–163. doi:10.1146/annurev-pathol-011110-130158
2. Wang H, Cui N, Su L, Long Y, Wang X, Zhou X, et al. Prognostic value of extravascular lung water and its potential role in guiding fluid therapy in septic shock after initial resuscitation. J Crit Care. 2016;33: 106–113. doi:10.1016/j.jcrc.2016.02.011
3. Arentz M, Yim E, Klaff L, Lokhandwala S, Riedo FX, Chong M, et al. Characteristics and Outcomes of 21 Critically Ill Patients With COVID-19 in Washington State. JAMA. 2020. doi:10.1001/jama.2020.4326
4. Gröger M, Pasteiner W, Ignatyev G, Matt U, Knapp S, Atrasheuskaya A, et al. Peptide Bbeta(15-42) preserves endothelial barrier function in shock. PloS One. 2009;4: e5391. doi:10.1371/journal.pone.0005391
5. Atar D, Petzelbauer P, Schwitter J, Huber K, Rensing B, Kasprzak JD, et al. Effect of intravenous FX06 as an adjunct to primary percutaneous coronary intervention for acute ST-segment elevation myocardial infarction results of the F.I.R.E. (Efficacy of FX06 in the Prevention of Myocardial Reperfusion Injury) trial. J Am Coll Cardiol. 2009;53: 720–729. doi:10.1016/j.jacc.2008.12.017
6. Wolf T, Kann G, Becker S, Stephan C, Brodt H-R, de Leuw P, et al. Severe Ebola virus disease with vascular leakage and multiorgan failure: treatment of a patient in intensive care. Lancet Lond Engl. 2015;385: 1428–1435. doi:10.1016/S0140-6736(14)62384-9
7. Krenn K, Lucas R, Croizé A, Boehme S, Klein KU, Hermann R, et al. Inhaled AP301 for treatment of pulmonary edema in mechanically ventilated patients with acute respiratory distress syndrome: a phase IIa randomized placebo-controlled trial. Crit Care Lond Engl. 2017;21: 194. doi:10.1186/s13054-017-1795-x
8. Tagami T, Ong MEH. Extravascular lung water measurements in acute respiratory distress syndrome: why, how, and when? Curr Opin Crit Care. 2018;24: 209–215. doi:10.1097/MCC.0000000000000503
9. Erban JK, Wagner DD. A 130-kDa protein on endothelial cells binds to amino acids 15-42 of the B beta chain of fibrinogen. J Biol Chem. 1992;267: 2451–2458.
10. Bach TL, Barsigian C, Yaen CH, Martinez J. Endothelial cell VE-cadherin functions as a receptor for the beta15-42 sequence of fibrin. J Biol Chem. 1998;273: 30719–30728.
11. Bergt S, Gruenewald M, Beltschany C, Grub A, Neumann T, Albrecht M, et al. The Fibrin-Derived Peptide Bβ15-42 (FX06) Ameliorates Vascular Leakage and Improves Survival and Neurocognitive Recovery: Implications From Two Animal Models of Cardiopulmonary Resuscitation. Crit Care Med. 2016;44: e988-995. doi:10.1097/CCM.0000000000001860
12. Roesner JP, Petzelbauer P, Koch A, Mersmann J, Zacharowski PA, Boehm O, et al. The fibrin-derived peptide Bbeta15-42 is cardioprotective in a pig model of myocardial ischemia-reperfusion injury. Crit Care Med. 2007;35: 1730–1735. doi:10.1097/01.CCM.0000269035.30231.76
13. Roesner JP, Petzelbauer P, Koch A, Tran N, Iber T, Mutz C, et al. A double blind, single centre, sub-chronic reperfusion trial evaluating FX06 following haemorrhagic shock in pigs. Resuscitation. 2009;80: 264–271. doi:10.1016/j.resuscitation.2008.10.019
14. Roesner JP, Petzelbauer P, Koch A, Tran N, Iber T, Vagts DA, et al. Bbeta15-42 (FX06) reduces pulmonary, myocardial, liver, and small intestine damage in a pig model of hemorrhagic shock and reperfusion. Crit Care Med. 2009;37: 598–605. doi:10.1097/CCM.0b013e3181959a12
15. De Backer D, Bakker J, Cecconi M, Hajjar L, Liu DW, Lobo S, et al. Alternatives to the Swan-Ganz catheter. Intensive Care Med. 2018;44: 730–741. doi:10.1007/s00134-018-5187-8
16. Bréchot N, Luyt C-E, Schmidt M, Leprince P, Trouillet J-L, Léger P, et al. Venoarterial extracorporeal membrane oxygenation support for refractory cardiovascular dysfunction during severe bacterial septic shock. Crit Care Med. 2013;41: 1616–1626. doi:10.1097/CCM.0b013e31828a2370
17. Caironi P, Tognoni G, Masson S, Fumagalli R, Pesenti A, Romero M, et al. Albumin replacement in patients with severe sepsis or septic shock. N Engl J Med. 2014;370: 1412–1421. doi:10.1056/NEJMoa1305727

18. Wu C, Chen X, Cai Y, Xia J, Zhou X, Xu S, et al. Risk Factors Associated With Acute Respiratory Distress Syndrome and Death in Patients With Coronavirus Disease 2019 Pneumonia in Wuhan, China. JAMA Intern Med. 2020. doi:10.1001/jamainternmed.2020.0994

18 LIST OF ADDENDA

Every addendum and the log of addenda versions are attached, independently of the protocol. Every addendum can be modified (change of addendum version) without modifying the version of the protocol.

18.1 List of investigators

18.2 Serious Adverse Events notification form

18.3 Pregnancy notification form

18.4 Investigator's Brochure

18.5 Immunogenicity – Final report
