# Supplementary material for: FX06 to rescue SARS-CoV-2-induced acute respiratory distress syndrome: a randomized clinical trial
Source: Crit Care. 2023 Aug 29;27:331. doi: 10.1186/s13054-023-04616-1 (PMC10463389; doi:10.1186/s13054-023-04616-1)

Online Supplementary Information

FX06 to rescue SARS-CoV-2–induced acute respiratory distress syndrome: a randomized clinical trial

Emmanuelle Guérin[1,2]; Lisa Belin[3]; Guillaume Franchineau[4,5]; Loïc Le Guennec[6,7]; David Hajage[3]; Mamadou Hassimiou Diallo[3]; Thomas Frapard[1,2]; Lucie Le Fèvre[1]; Charles-Edouard Luyt[1,8]; Alain Combes[1,8]; Stéphane Germain[2]; Jan Hayon[4]; Pierre Asfar[9] and Nicolas Bréchot[1,2,10,11]

[1]Service de Médecine Intensive-Réanimation, Institut de Cardiologie, Assistance Publique–Hôpitaux de Paris (APHP), Hôpital Pitié–Salpêtrière, Paris, France. [2]Center for Interdisciplinary Research in Biology, Collège de France, Centre National de la Recherche Scientifique, Institut National de la Santé et de la Recherche Médicale (INSERM), Université PSL, Paris, France. [3]Sorbonne Université, INSERM, Institut Pierre Louis d'Epidémiologie et de Santé Publique, AP-HP, Hôpital Pitié Salpêtrière, Département de Santé Publique, Unité de Recherche Clinique PSL-CFX , CIC-1901, F75013, Paris, France. [4]Intensive Care Unit, Centre Hospitalier Intercommunal de Poissy–Saint-Germain-en-Laye, Poissy, France. [5]INSERM U1018, Centre de Recherche en Épidémiologie et Santé Des Populations (CESP), Equipe "Rein et Cœur", Université Paris Saclay, Villejuif, France. [6]Médecine Intensive-Réanimation Neurologique, Hôpital Pitié–Salpêtrière, APHP, Paris, France. [7]Sorbonne Universités, Paris, France. [8]Sorbonne Université INSERM-UMRS 1166, Institute of Cardiometabolism and Nutrition, Paris, France. [9]Service de Médecine Intensive-Réanimation et Médecine Hyperbare, Centre Universitaire Hospitalier d'Angers, Angers, France. [10]Service de Médecine Intensive-Réanimation, Hôpital Européen Georges-Pompidou, APHP, Paris, France. [11]Université Paris Cité, Paris, France.

Corresponding author: Nicolas Bréchot, Service de Médecine Intensive-Réanimation, Hôpital Européen Georges-Pompidou, Paris, France. Phone: +33 1 56 09 23 42; Fax: +33 1 56 09 32 02; e-mail: nicolas.brechot@aphp.fr

TABLE of CONTENTS

SUPPLEMENTARY METHODS **3**
Preliminary study on transpulmonary thermodilution measurements in patients on venovenous-ECMO 3

RATIONAL FOR DOSE SELECTION **7**

SUPPLEMENTARY REFERENCES **8**

SUPPLEMENTARY FIGURES **9**
Figure S2. Thermodilution-measured EVLWi kinetics from day 1 (randomization) to day 7 for each group. 9
Figure S3. Individual and mean EVLWis from day 1 (randomization) to day 7 for each group 10
Figure S4. Kinetics of thermodilution measured pulmonary vascular permeability index from day 1 (randomization) to day 7 for each group. 11
Figure S5. Twenty-four hour fluid balance from day 1 (randomization) to day 7 for each group 12
Figure S6. PaO_2/FiO_2-ratio kinetics from day 1 (randomization) to day 7 for each group 13
Figure S7. Weinberg Radiological Score kinetics from day 1 (randomization) to day 30 for both groups: (A) before and (B) after imputation for missing data. 14
Figure S8. Invasive blood pressure kinetics from day 1 (randomization) to day 7 for both groups 15
Figure S9. SOFA (Sequential Organ Failure Assessment) score kinetics from day 1 (randomization) to day 15 for both groups 16
Figure S10. Sixty-day survival for each group 17

ONLINE METHODS

Preliminary Study on Transpulmonary Thermodilution Measurements in Patients on Venovenous (VV)-ECMO

COVID-19 patients admitted in intensive care units are particularly susceptible to benefit from rescue therapy with VV-ECMO. Because high ECMO blood flow might impact measurements of several parameters during transpulmonary thermodilution, including the extravascular lung-water index (EVLWi) [1,2], we conducted a preliminary study to guide our protocol measurements for those patients. From April 30, 2020, to September 30, 2021, transpulmonary thermodilution measurements were obtained for all patients with both thermodilution catheters and on VV-ECMO, and referred for ECMO explantation, during a gradual decrease of ECMO blood flow from 5 L/min to 0 L/min (ECMO clamped). VV-ECMO cannulation used femoral–right jugular lines, and the venous thermodilution catheter was inserted into the left internal jugular vein of all patients. Parameters were averaged after 2 injections of cold saline solution, and indexed to each patient's predicted body weight. A French Society of Intensive Care Medicine (SRLF) Ethics Committee approved the study.

Results

Twenty patients were included in the study. Their characteristics at the time of ECMO explantation are reported in Table S1.

Table S1. Characteristics of the 20 preliminary study participants at baseline

Characteristic	Value*
Age, y	47 [40–54]
Sex,	
Male	16 (80)
Female	4 (20)
Body mass index, kg/m^2	34.1 [30.9–42.2]
Preexisting conditions	
Diabetes	6 (30)
Arterial Hypertension	8 (40)
Coronary artery disease	0
Chronic respiratory disease	0
Kidney disease†	0
Chronic liver disease	0
Immunodeficiency	0
Cancer	2 (10)
Charlson Comorbidity Score	1 [0–1]
McCabe and Jackson severity score	1 [1–1]
Days to enrollment from	
Symptom onset	36.5 [19.8–52.3]
Mechanical ventilation onset	22.5 [9.3–40.0]
VV-ECMO initiation	18.0 [18.0–34.5]
SOFA score at enrollment	7 [6–9]
COVID-19 treatments administered	16 (80)
Corticosteroids	15 (75)

Remdesivir	2 (10)
Immunoglobulins	0
Tocilizumab	0
Other	0
Respiratory status	
Invasive mechanical ventilation	20 (100)
Tidal volume, mL	400 [350–420]
Positive end-expiratory pressure, cmH_2O	12 [8–14]
Plateau pressure, cmH_2O	28 [25–30]
Arterial blood gases	
Arterial pH	7.5 [7.4–7.5]
PaO_2/FiO_2, mmHg	221 [167–265]
$PaCO_2$, mmHg	41.5 [37.2–46.0]
Weinberg Radiological Score	7.5 [6.0–9.8]
Vasopressors	2 (10)
Laboratory analyses	
White blood cell count, G/L	14.7 [11.8 -17.9]
Hemoglobin level, g/dL	7.7 [7.1–8.3]
Platelet count, G/L	216 [154–263]
SGOT (ASAT), IU/L	43 [32–70]
SGPT (ALAT), IU/L	48 [24–72]
Creatine phosphokinase, IU/L	124 [46–459]
QT ratio, %	80 [76–85]
Activated partial thromboplastin time ratio	1.5 [1.4–1.8]
Fibrinogen, g/L	3.9 [1.8–5.9]
D-Dimers, µg/mL	20000 [8680–20000]
Serum albumin, g/L	23 [20–24]
N-terminal pro-brain natriuretic peptide, pg/mL	54 [36–179]

*Values are expressed as number (%) or median [25th–75th percentiles; IQR]. *SOFA* Sequential Organ-Failure Assessment; *VV-ECMO* venovenous extracorporeal membrane oxygenation.
†Determined from the most recent stable serum creatinine level prior to this hospital admission, except for patients on dialysis. Abnormal kidney function was defined as creatinine at ≥130 µmol/L (≥1.5 mg/dL) for males or ≥100 µmol/L (≥1.1 mg/dL) for females not previously on dialysis.

Although extra-vascular lung water index (EVLWi) and pulmonary vascular permeability index (PVPI) measurements were significantly affected by high blood flows on the ECMO system, they showed minimal variations compared to their measurements without ECMO for blood flows <2 L/min (Figure S1). Bland–Altman analysis revealed adequate agreement between EVLWi measurements with ECMO blood flows at 2 or 1 L/min, and its measurement at 0 L/min, taken as the reference method (respective mean±SD [95% limits of agreement] biases of –0.21± 0.68 [–1.55;1.12] and –0.16±0.59, [–1.32;0.99] mL/kg). ECMO blood flows up to 5 L/min did not affect the determination of cardiac index in that study.

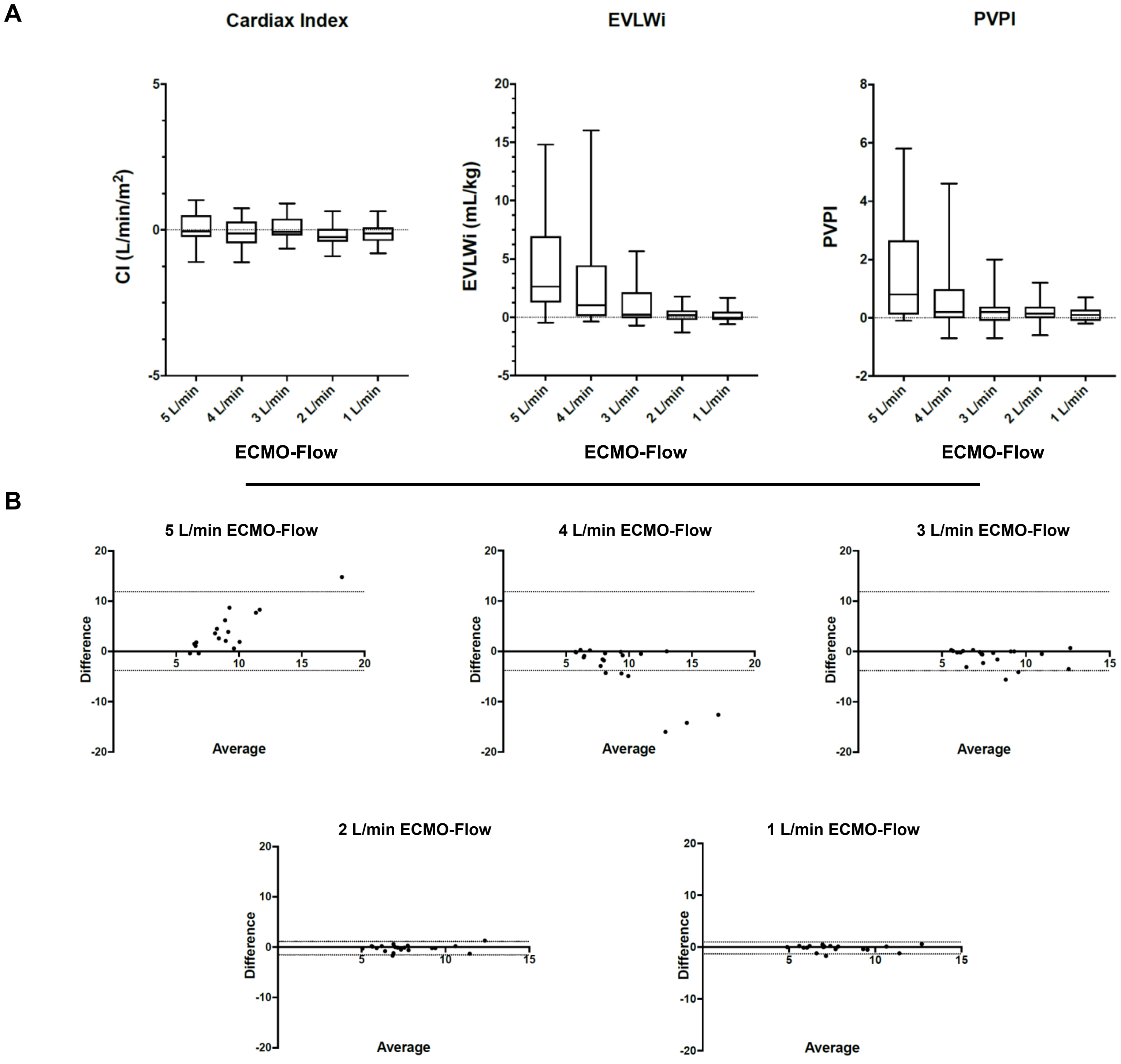

Figure S1. Influence of VV-ECMO blood flow on transpulmonary thermodilution measurements of cardiac index, EVLWi and PVPI. (A) Box plots of the differences between measurements at each ECMO blood flow and that obtained with the ECMO blood flow at 0 L/min. Internal horizontal line is the median, the lower and upper box limits are the 25th and 75th percentiles, and T-bars represent the range. **(B)** Bland–Altman analyses of the agreement between EVLWi measurements at various ECMO blood flows, and its determination with ECMO blood flow at 0 L/min. *EVLWi* extravascular lung-water index; *PVPI* pulmonary vascular permeability index.

Interpretation Based on this preliminary study, together with data from another group in 7 patients explanted from VV-ECMO [2], we concluded that transpulmonary thermodilution EVLWi determination was reliable during VV-ECMO, provided that ECMO blood flow was reduced to ≤2 L/min. Hence, patients on VV-ECMO were not excluded from the trial, but investigators were asked to transiently lower ECMO blood flow to ≤2 L/min for those patients at the time of transpulmonary thermodilution measurements. All parameters could be determined at all time points using this protocol for trial patients on VV-ECMO.

RATIONALE FOR DOSE SELECTION

The FX06 dose–response was first evaluated in a murine model of myocardial ischemia–reperfusion, with doses up to 100 mg/kg [3]. No additional myocardium-protection improvement was observed above 2.4 mg/kg. The FX06 effect at this dose during ischemia–reperfusion was further validated in a pig model of myocardial ischemia [4] and a murine model of cardiac arrest [5]. During ARDS, an escalating-dose study was conducted using a mouse model of dengue-virus infection, with doses ranging from 0.6 to 9.6 mg/kg/d i.p. Similar effects on survival were found for the last 2 doses—4.8 and 9.6 mg/kg/d—with markedly reduced vascular leakage in the lungs confirmed for the 4.8 mg/kg/d dose on days 5 and 7 of infection, as assessed with Evans blue-dye extravasation [6]. Therefore, FX06 was initially used at the dose of 400 mg/d (close to 4.8 mg/kg/d for a normal weight patient) for 3 days, as rescue therapy for a patient with severe Ebola-induced ARDS. Started on day 11, its use was associated with sharply decreased EVLWi [7]. Furthermore, the results of safety studies strongly supported good FX06 tolerance at doses far above that range. A dose up to 240 mg/kg was well-tolerated in dogs, with only mild and transient hypotension [4]. A phase I trial in healthy humans found no adverse event for doses up to 17.5 mg/kg/d [4]. Finally, no safety issue was detected when FX06 at 400 mg/d was repeatedly injected for 4– 7 days, as rescue therapy for severe COVID-19–associated ARDS [8]. The fixed dose of 400 mg/d for 5 days (close to 4.8 mg/kg/d) was finally retained for this study.

Supplementary References

1. Herner A, Lahmer T, Mayr U, Rasch S, Schneider J, Schmid RM, et al. Transpulmonary thermodilution before and during veno-venous extra-corporeal membrane oxygenation ECMO: an observational study on a potential loss of indicator into the extra-corporeal circuit. J Clin Monit Comput. 2020;34:923–36.

2. Loosen G, Conrad AM, Hagman M, Essert N, Thiel M, Luecke T, et al. Transpulmonary thermodilution in patients treated with veno-venous extracorporeal membrane oxygenation. Ann Intensive Care. 2021;11:101.

3. Petzelbauer P, Zacharowski PA, Miyazaki Y, Friedl P, Wickenhauser G, Castellino FJ, et al. The fibrin-derived peptide Bbeta15-42 protects the myocardium against ischemia-reperfusion injury. Nat Med. 2005;11:298–304.

4. Roesner JP, Petzelbauer P, Koch A, Mersmann J, Zacharowski PA, Boehm O, et al. The fibrin-derived peptide Bbeta15-42 is cardioprotective in a pig model of myocardial ischemia-reperfusion injury. Crit Care Med. 2007;35:1730–5.

5. Bergt S, Gruenewald M, Beltschany C, Grub A, Neumann T, Albrecht M, et al. The Fibrin-Derived Peptide Bβ15-42 (FX06) Ameliorates Vascular Leakage and Improves Survival and Neurocognitive Recovery: Implications From Two Animal Models of Cardiopulmonary Resuscitation. Crit Care Med. 2016;44:e988-995.

6. Gröger M, Pasteiner W, Ignatyev G, Matt U, Knapp S, Atrasheuskaya A, et al. Peptide Bbeta(15-42) preserves endothelial barrier function in shock. PloS One. 2009;4:e5391.

7. Wolf T, Kann G, Becker S, Stephan C, Brodt H-R, de Leuw P, et al. Severe Ebola virus disease with vascular leakage and multiorgan failure: treatment of a patient in intensive care. Lancet Lond Engl. 2015;385:1428–35.

8. Adam EH, Schmid B, Sonntagbauer M, Kranke P, Zacharowski K, Meybohm P. Fibrin-derived peptide Bβ15-42 (FX06) as salvage treatment in critically ill patients with COVID-19-associated acute respiratory distress syndrome. Crit Care. 2020;24:574.

SUPPLEMENTARY FIGURES

Figure S2. Thermodilution-measured EVLWi kinetics from day 1 (randomization) to day 7. Mean (SD) are shown for each group. *EVLWi* extravascular lung-water index. Adjusted mean change and ANCOVA significance (p=0.57) indicates no EVLWi kinetic differences between groups.

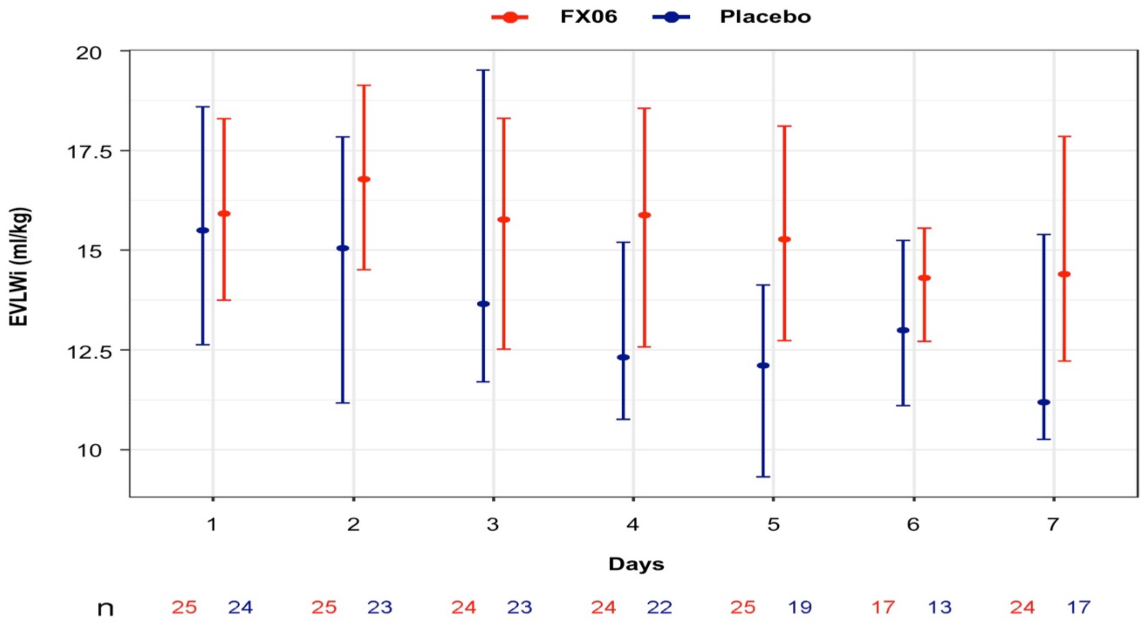

Figure S3. Individual and mean EVLWis from day 1 (randomization) to day 7 for each group. *EVLWi* extravascular lung-water index. Mann–Whitney *U*-test (pre-planned sensitivity analysis) comparisons of the D7 – D1 EVLWi changes between arms showed they did not differ significantly (*p*=0.51).

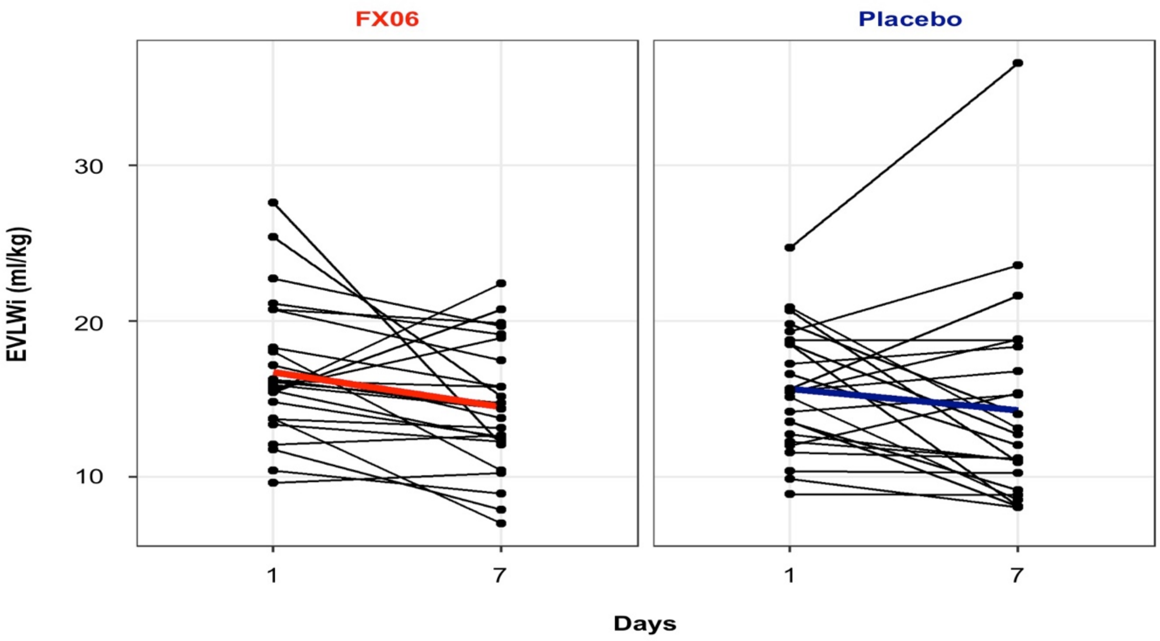

Figure S4. Thermodilution-measured pulmonary vascular permeability index (PVPI) kinetics of from day 1 (randomization) to day 7 for each group. Means (SD) are shown for each group. Linear mixed model p=0.55.

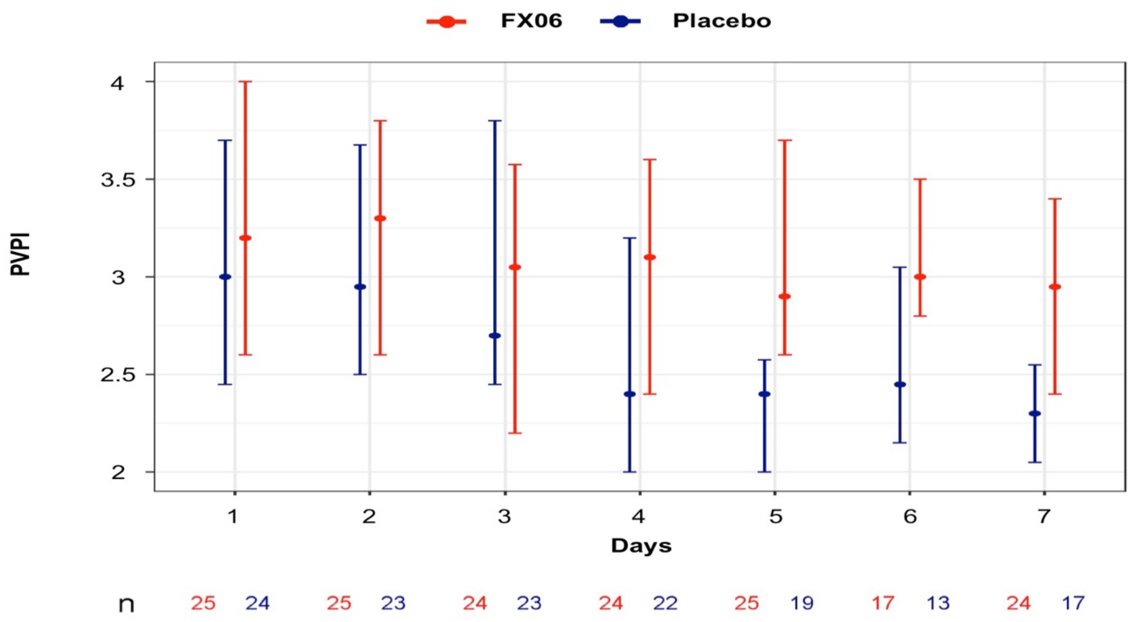

Figure S5. Twenty-four hour fluid balance from day 1 (randomization) to day 7 for each group. Tukey's Box plots are presented. Linear mixed model *p*=0.27.

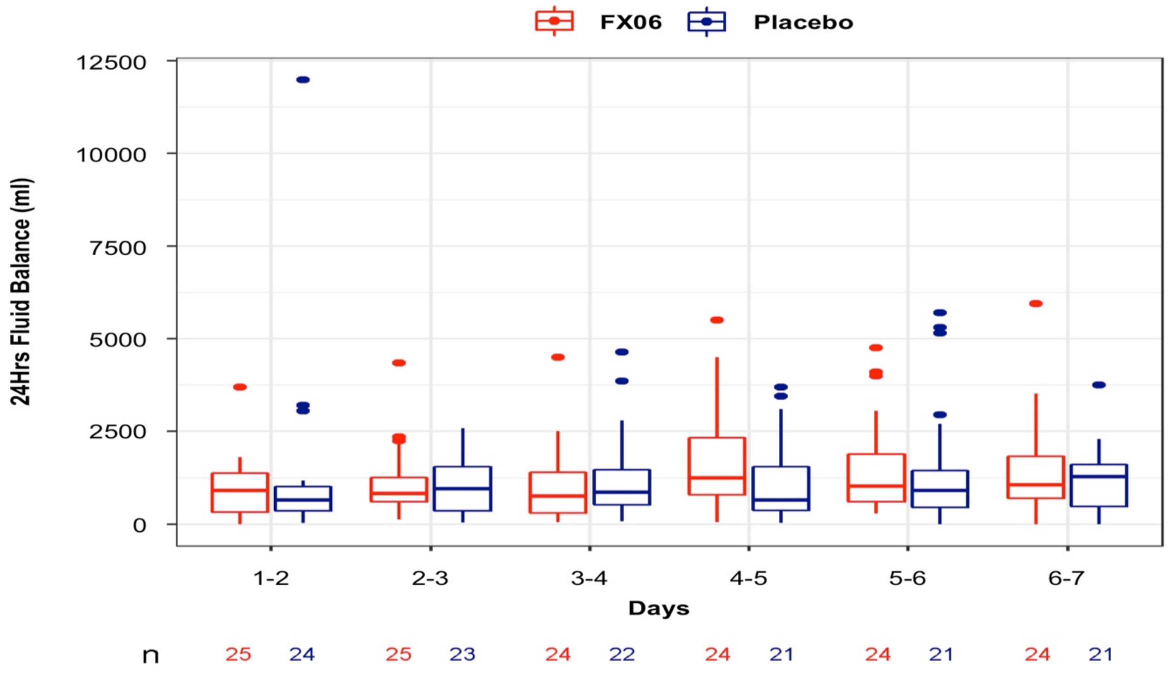

Figure S6. PaO_2/FiO_2-ratio kinetics from day 1 (randomization) to day 15 for each group. Means (SD) are shown. Linear mixed model p=0.39.

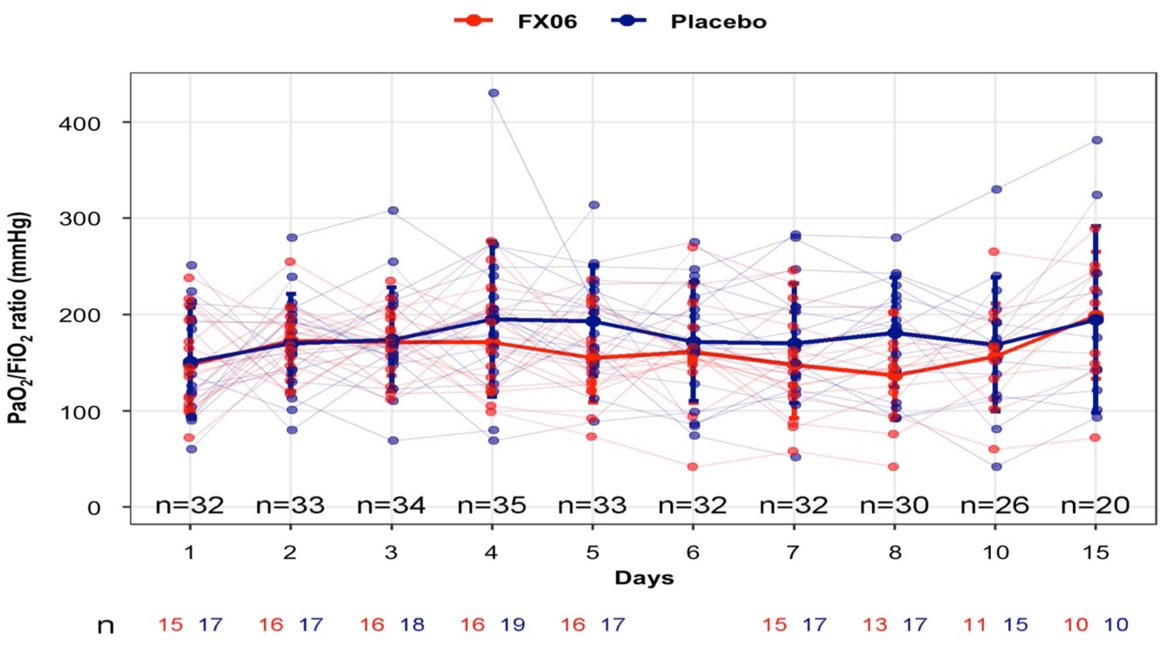

Figure S7. Weinberg Radiological Score kinetics from day 1 (randomization) to day 30 for both groups: (A) before and (B) after imputation for missing data. Means (SD) are shown. P-values from linear mixed models indicate that Radiological Weinberg Score kinetics could differ between groups before imputation for missing data (p<0.001) but not after (p=0.20).

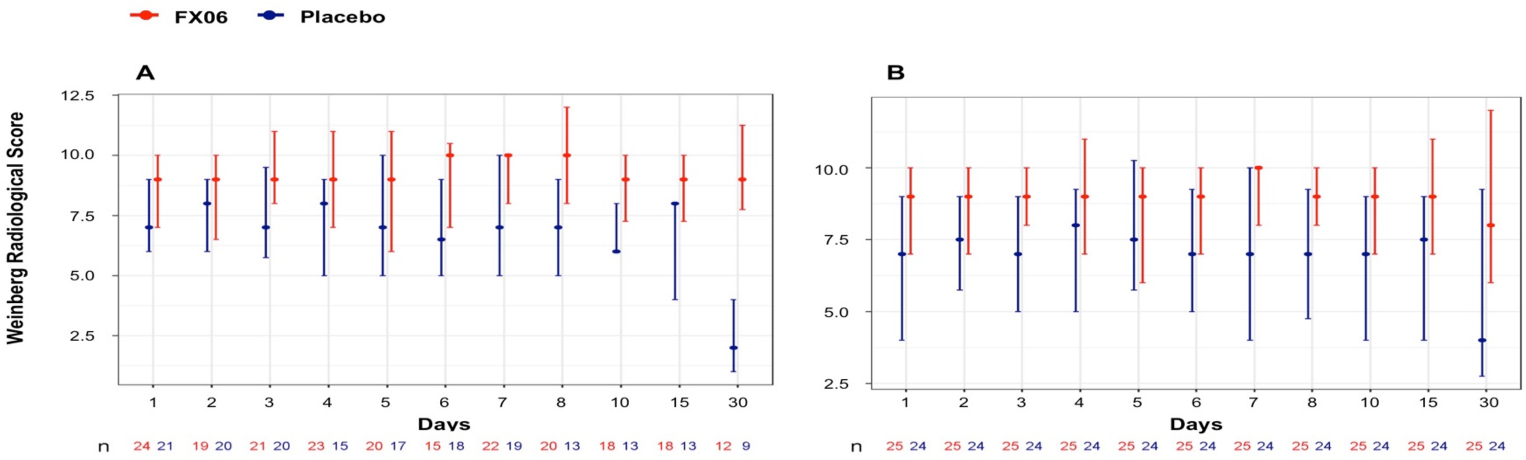

Figure S8. Invasive blood-pressure kinetics from day 1 (randomization) to day 7 for both groups. Means (SD) are shown for each group. P-values from linear mixed models indicate that invasive blood-pressure kinetics differed between groups: respectively *p*=0.01, *p*=0.01 and *p*=0.02 for comparisons of mean, systolic and diastolic blood pressures.

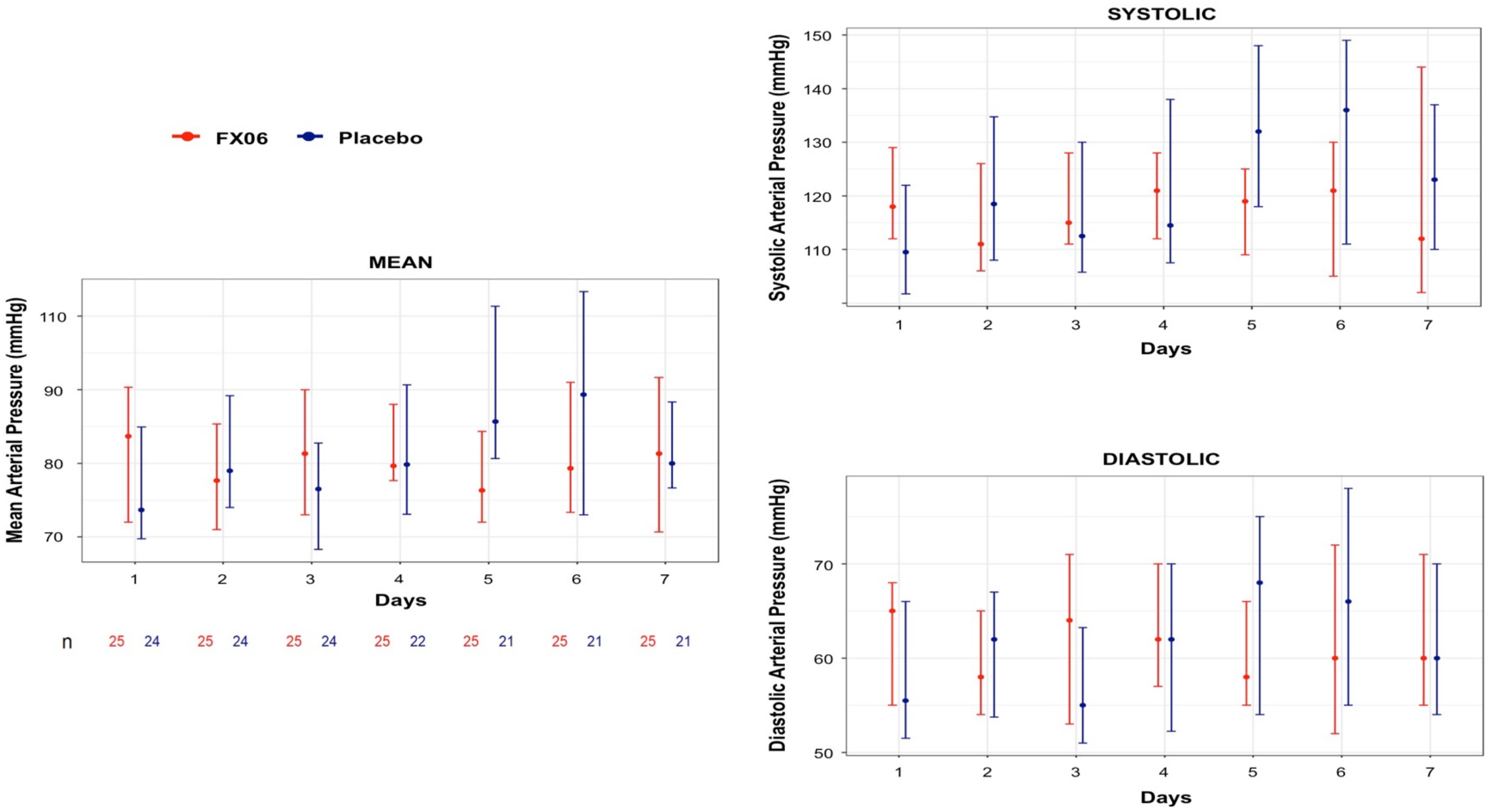

Figure S9. SOFA (Sequential Organ-Failure Assessment) score kinetics, from day 1 (randomization) to day 15 for both groups. Means (SD) are shown. Linear mixed model p=0.36.

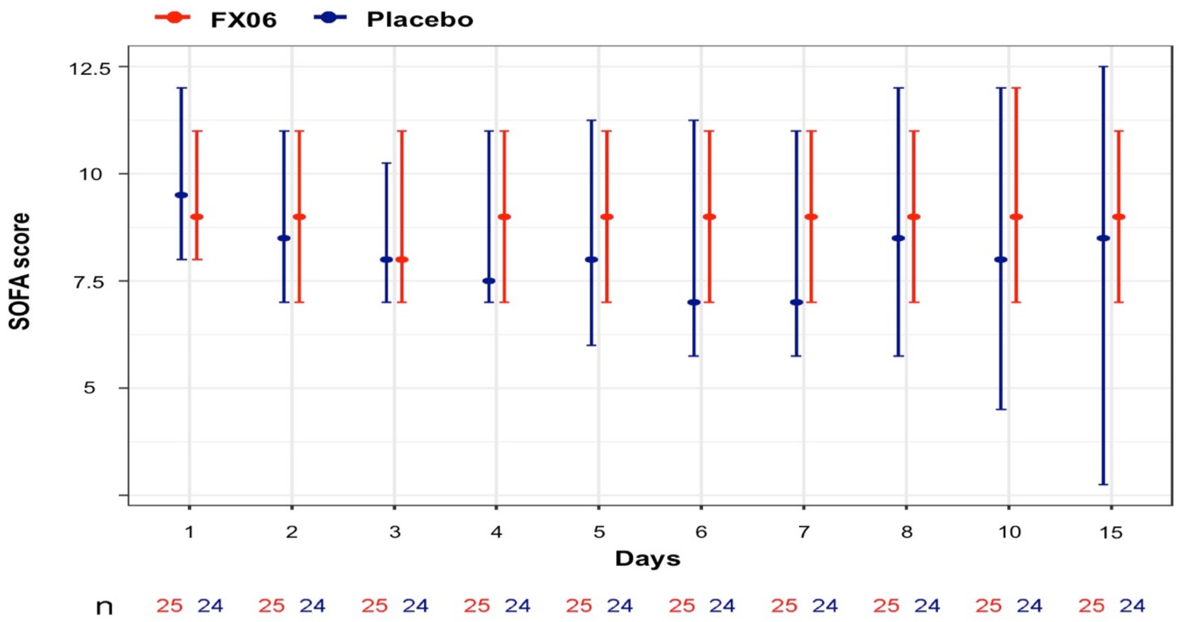

Figure S10. Sixty-day survival for each group.

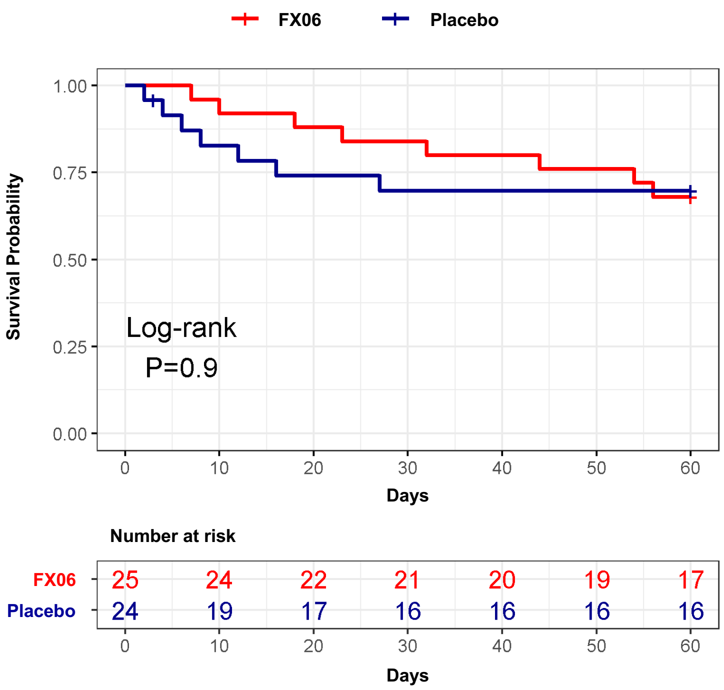

Supplement: Supplementary file 2 — Additional file 2. The supplementary data. [file 13054_2023_4616_MOESM2_ESM.pdf]
